# Atlas of *Plasmodium falciparum* intraerythrocytic development using expansion microscopy

**DOI:** 10.1101/2023.03.22.533773

**Published:** 2023-05-15

**Authors:** Benjamin Liffner, Ana Karla Cepeda Diaz, James Blauwkamp, David Anaguano, Sonja Frölich, Vasant Muralidharan, Danny W. Wilson, Jeffrey Dvorin, Sabrina Absalon

**Affiliations:** 1 Department of Pharmacology and Toxicology, Indiana University School of Medicine, Indianapolis, IN, USA.; 2 Biological and Biomedical Sciences, Harvard Medical School, Boston MA, USA.; 3 Division of Infectious Diseases, Boston Children’s Hospital, Boston MA, USA.; 4 Center for Tropical and Emerging Global Diseases, University of Georgia, Athens, GA, USA.; 5 Department of Cellular Biology, Franklin College of Arts and Sciences, University of Georgia, Athens, GA, USA.; 6 Research Centre for Infectious Diseases, School of Biological Sciences, University of Adelaide, Adelaide, SA, Australia.; 7 Institute for Photonics and Advanced Sensing, University of Adelaide, Adelaide, SA, Australia.; 8 Burnet Institute, 85 Commercial Road, Melbourne, VIC, Australia.; 9 Department of Pediatrics, Harvard Medical School, Boston, MA, USA.

## Abstract

Apicomplexan parasites exhibit tremendous diversity in much of their fundamental cell biology, but study of these organisms using light microscopy is often hindered by their small size. Ultrastructural expansion microscopy (U-ExM) is a microscopy preparation method that physically expands the sample ~4.5x. Here, we apply U-ExM to the human malaria parasite *Plasmodium falciparum* during the asexual blood stage of its lifecycle to understand how this parasite is organized in three-dimensions. Using a combination of dye-conjugated reagents and immunostaining, we have catalogued 13 different *P. falciparum* structures or organelles across the intraerythrocytic development of this parasite and made multiple observations about fundamental parasite cell biology. We describe that the microtubule organizing center (MTOC) and its associated proteins anchor the nucleus to the parasite plasma membrane during mitosis. Furthermore, the rhoptries, Golgi, basal complex, and inner membrane complex, which form around this anchoring site while nuclei are still dividing, are concurrently segregated and maintain an association to the MTOC until the start of segmentation. We also show that the mitochondrion and apicoplast undergo sequential fission events while maintaining an MTOC association during cytokinesis. Collectively, this study represents the most detailed ultrastructural analysis of *P. falciparum* during its intraerythrocytic development to date, and sheds light on multiple poorly understood aspects of its organelle biogenesis and fundamental cell biology.

## INTRODUCTION

**Figure 1:**
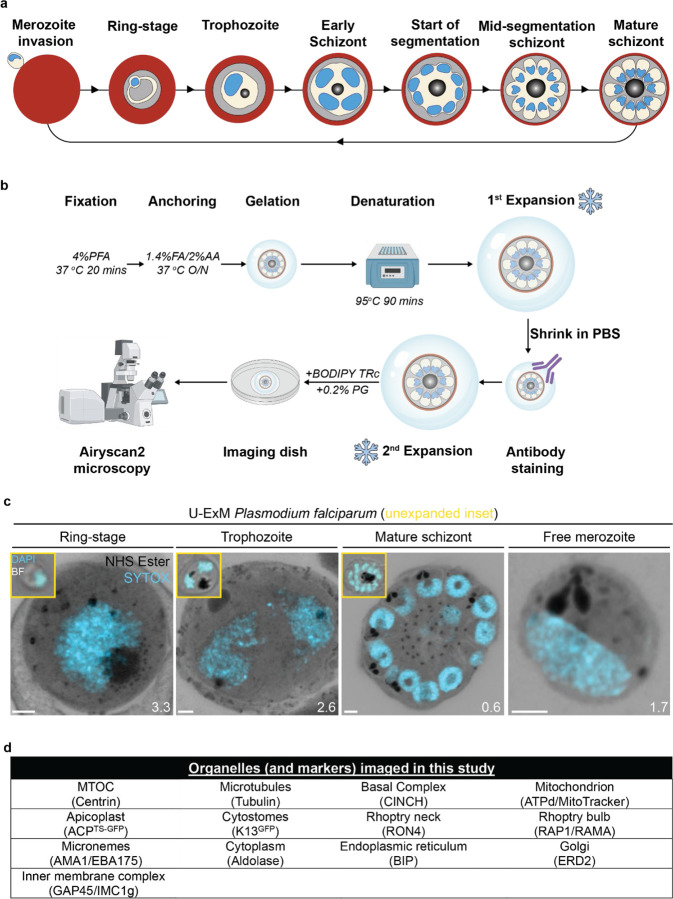
U-ExM workflow and summary of parasite structures imaged in this study. **(a)** Asexual blood stage lifecycle of P. falciparum. **(b)** Ultrastructure expansion microscopy (U-ExM) workflow used in this study. PFA = paraformaldehyde, FA = formaldehyde, AA = acrylamide PG = propyl gallate. Snowflake indicates steps where gels were cryopreserved. **(c)** Comparison of brightfield and DAPI staining of unexpanded P. falciparum parasites (inset) with P. falciparum prepared by U-ExM, stained with NHS Ester (protein density; greyscale) and SYTOX Deep Red (DNA; cyan) and imaged using Airyscan microscopy. Images are maximum-intensity projections, number on image = Z-axis thickness of projection in µm. Scale bars = 2 µm. **(d)** Summary of all organelles, and their corresponding antibodies, imaged by U-ExM in this study.

The human malaria parasite *Plasmodium falciparum* has a complex lifecycle that involves both human and mosquito hosts. Of its many lifecycle stages, the asexual replication of *P. falciparum* inside human red blood cells (RBCs) is responsible for the clinical symptoms of malaria. This asexual blood stage starts when a merozoite invades a host RBC and transitions through several morphologies before forming approximately 30 new daughter merozoites which egress from their host cell and invade new RBCs (Figure 1a). Host RBCs are approximately 7–8 µm in diameter^1^ and contain dozens of parasites, each with their own sets of organelles and structures. The small size of *P. falciparum* and its organelles still poses a challenge to the study of many facets of *P. falciparum* cell biology, especially when immunostaining is required.

Expansion microscopy, which has gained popularity over the last decade, is a set of sample preparation techniques that isotropically increase the physical size of a microscopy sample^2^. While many expansion microscopy methods have been developed, ultrastructure expansion microscopy (U-ExM)^3^ was the first used in *Plasmodium* and since has been used in *P. falciparum* and across multiple apicomplexan parasites^4–9^. U-ExM results in the ~4.5 fold isotropic expansion of the sample and largely preserves its proteome, making it compatible with antibody staining and many fluorescent dyes^3^. The increase in physical sample size results in a dramatic increase in the ability to identify and distinguish different parasite structures. Thus, some structures that could previously only be investigated using electron microscopy can now be studied with the flexibility, scalability, and inexpensive nature of conventional light microscopy.

Application of U-ExM to *Plasmodium*, and other Apicomplexa, has already enhanced our understanding of parasite cell biology tremendously, resulting in the identification of new parasite structures and better characterization of the size and shape of others^4–6,10–13^. Its significant impact on the field in such a short amount of time indicates U-ExM will be a technique heavily used in both *Plasmodium* and Apicomplexa more broadly for the foreseeable future. Considering this, we set out to image *P. falciparum* structures and organelles across the asexual blood stage of the lifecycle to serve as a reference for the expanding number of U-ExM users who study Apicomplexa and to uncover previously invisible aspects of the cell biology of *P. falciparum*.

## RESULTS

### Ultrastructure Expansion Microscopy (U-ExM) reveals multiple parasite structures without the use of antibodies.

Dyes that are not antigen-specific are commonplace in light microscopy. N-hydroxysuccinimide (NHS) esters conjugated to dyes are amino-reactive and can be used for fluorescent labelling of protein density^14^. Similarly, BODIPY TR ceramide (BODIPY TRc) and other dye-conjugated fatty acids are commonly used for labelling lipids^15^. Coupling U-ExM with these dyes has already revealed parasite structures for which specific antibodies did not exist^5,6,10^. While antibody-based labelling provides high-specificity, this labelling lacks complexity and is limited to the specific protein or antigen that is being targeted. The more general stains increase the number of parasite features or organelles we can observe in the same sample without additional antibody markers. Therefore, these general stains allow for low-specificity but high-complexity imaging, similar to the use of uranyl acetate in electron microscopy^16^. To better profile the subcellular organization of *P. falciparum* during the asexual blood stages, we set out to determine what parasite structures could, and could not, be visualised by U-ExM when using some of these dyes.

We, and others, have previously shown that combining BODIPY TRc and dye-conjugated NHS ester with U-ExM allows visualisation of the parasite plasma membrane (PPM), parasitophorous vacuolar membrane (PVM), nuclear envelope, rhoptries, endoplasmic reticulum, microtubule organizing center (MTOC), basal complex, and apical polar rings (APRs)^5,6,10^. However, many important organelles and parasite structures are either not identifiable using these stains or have yet to be validated, including the mitochondrion, apicoplast, and cytostomes.

To identify and validate the location of as many parasite organelles and structures as possible, we utilized U-ExM coupled with BODIPY TRc, Alexa Fluor 405-conjugated NHS ester (which we will refer to as “NHS ester”), the nucleic acid (i.e., DNA) stain SYTOX Deep Red, and antibodies directed against 13 different subcellular targets (microtubules, MTOC, basal complex, IMC, mitochondrion, apicoplast, cytostome, rhoptry bulb, rhoptry neck, micronemes, cytoplasm, endoplasmic reticulum, and Golgi). Parasites were harvested at multiple time points during the intraerythrocytic asexual stage and imaged using Airyscan2 super-resolution microscopy, providing high-resolution three-dimensional imaging data (Figure 1c). A full summary of all target-specific stains used in this study can be found in Figure 1d.

The first protein we imaged was aldolase, a marker of the parasite cytoplasm (Supplementary Figure 1). Aldolase staining was present in all asexual replication stages. During the ring stage, the “ameboid” shape of the parasite is readily visualized, consistent with previous studies of this stage in time-lapse microscopy of live parasites^17^ (Supplementary Figure 1). Regions within the parasite where both aldolase and NHS ester staining were absent are consistent with the expected area of the food vacuole. Typically, the food vacuole would be filled with hemozoin, however, this crystal likely cannot expand and therefore leaves a large space inside the parasite that does not contain significant protein density (Supplementary Figure 1).

### The microtubule organizing center (MTOC) and microtubules.

The first major transition during the blood stage of the lifecycle occurs when the parasites turn from rings into trophozoites. Soon after this transition, the parasite will begin to replicate its DNA and undergo mitosis followed by nuclear fission^18^. Mitosis is coordinated by microtubules, which are in turn nucleated by structures called microtubule organizing centers (MTOCs)^19^. It was recently shown that the nuclear MTOC which coordinates *P. falciparum* mitosis is comprised of intranuclear and cytoplasmic portions^10^. The most commonly used MTOC markers are the centrins, which in *Plasmodium* comprise four proteins that localize to the cytoplasmic portion of the MTOC and appear after intranuclear microtubules have already formed ^10^. Given that the MTOC is required for microtubule formation, this implies that the MTOC forms before centrin is visible. To investigate these processes in more detail, we visualized the biogenesis and dynamics of the MTOC and microtubules during the trophozoite and schizont stages by pairing NHS ester, which we have recently shown can stain both the intranuclear and cytoplasmic portions of the MTOC^6^, with an anti-centrin antibody (Clone 20H5, raised against centrin from *Chlamydomonas*). This antibody likely recognizes centrin 3 in *P. falciparum*^20^, but a recent study suggests that all four *P. falciparum* centrins share an MTOC localization^21^.

#### MTOC biogenesis and disassembly

**Figure 2:**
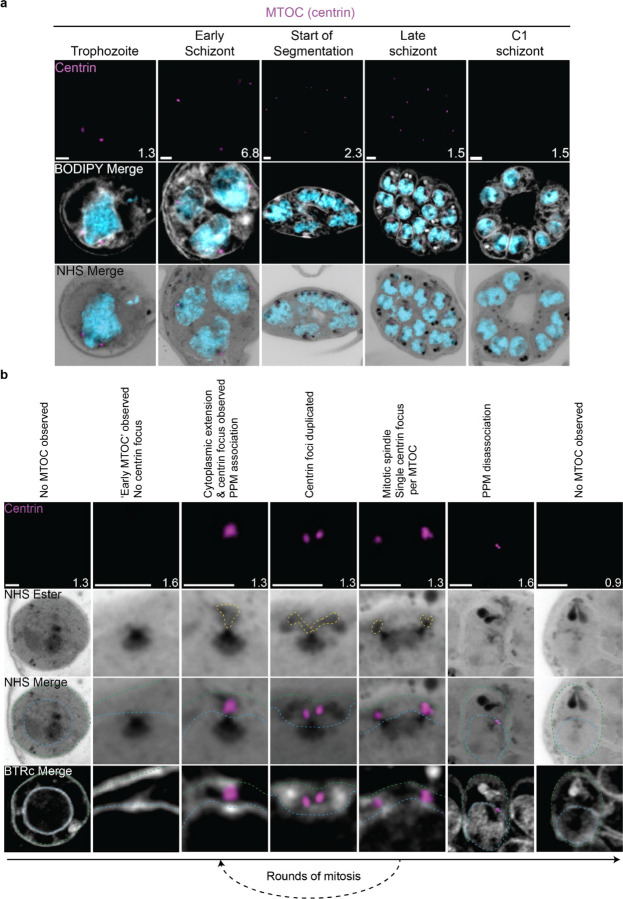
Microtubule organizing center (MTOC) biogenesis and dynamics. **(a)** 3D7 parasites were prepared by U-ExM, stained with NHS ester (greyscale), BODIPY TRc (white), SYTOX (cyan) and anti-centrin (MTOC; magenta) antibodies and imaged using Airyscan microscopy across the asexual blood stage. **(b)** Proposed timeline of events in MTOC biogenesis, dynamics, and disassembly. Yellow line = cytoplasmic extensions, blue line = nuclear envelope, green line = parasite plasma membrane. Images are maximum-intensity projections, number on image = Z-axis thickness of projection in µm. Scale bars = 2 µm.

Neither a recognizable MTOC nor above-background centrin staining were observed in ring-stage parasites (Figure 2b)^10^. The MTOC first appeared in mononucleated trophozoites but changed morphology as these parasites got closer to their first nuclear division. In the 23 mononucleated trophozoites we imaged, 52% of MTOCs lacked cytoplasmic extensions (Supplementary Figure 2a&b). These MTOCs only had their intranuclear portion, lacking cytoplasmic extensions as observed by NHS ester (Figure 2a&b). We will refer to this as the ‘early’ MTOC. All 12 of the ‘early’ MTOC trophozoites we imaged also lacked centrin staining (Supplementary Figure 2c). This matches previous reports that centrin is specifically associated with the cytoplasmic domain of the MTOC^10^. As expected, this ‘early’ MTOC was capable of nucleating microtubules (Supplementary Figure 2a). The centrin focus and cytoplasmic extensions became visible in mononucleated trophozoites after nucleation of the intranuclear microtubules but prior to the first MTOC duplication event (Supplementary Figure 2a), consistent with previous reports^10^. The cytoplasmic extensions of the MTOC began at the nuclear membrane and ended at an NHS ester-dense focus located at the parasite plasma membrane (PPM) (Figure 2b). This association between the nuclear MTOC and the PPM has previously been observed in gametocytes and asexual blood stages^22,23^, but the temporal nature of this association during the asexual blood stages remained uncharacterized. Our observation of cytoplasmic tethers by NHS ester, discussed further below, combined with the temporal pattern of MTOC-PPM association suggests that nuclei are physically anchored to the PPM while parasites are undergoing mitosis.

For as long as parasites continue to undergo mitosis throughout schizogony, all MTOC cytoplasmic extensions remain in contact with the PPM (Figure 2a). These cytoplasmic extensions showed one or two centrin foci that largely matched branch number. That is, of the 102 single-branched MTOCs observed, all had a single centrin focus, and of the 28 double-branched MTOCs observed, 92% had two centrin foci (Figure 2b; Supplementary Figure 2c). The overwhelming coordination between number of cytoplasmic extensions and number of centrin foci suggests centrin duplication and duplication of the cytoplasmic tethers of the MTOC happens quickly and simultaneously. The few cases when there is a mismatch between the number of cytoplasmic extensions and centrin foci all occur in double-branched MTOCs and can be attributed to limitations in our ability to resolve two centrin foci (Supplementary Figure 2c&d).

The relative abundance of nuclei with single MTOCS, defined as MTOCs lacking a mitotic spindle, versus mitotic MTOCs, MTOCs anchored to a mitotic spindle, varied throughout schizogony as did their branch numbers (Supplementary Figure 2). We hypothesize that MTOCs start out without any cytoplasmic extensions and then develop a single cytoplasmic extension and centrin focus which duplicate ahead of MTOC duplication and then segregate with each MTOC during miotic spindle formation and karyokinesis (Figure 2b). This means that during the rapid mitotic events of schizogony that take place at the 6–12 nuclei stage, single MTOCs with a single cytoplasmic extension are very rare. We observed them in just 6% of 221 imaged MTOCs (Supplementary Figure 2b). Single MTOCs with a single cytoplasmic extension were only abundant when the pace of nuclear replication was slow at the early schizont stage (24% of 76 MTOCs imaged in cells with 2–5nuclei) and the end of segmentation (44% of 290 MTOCs imaged in segmenting parasites).

This observed pattern of duplication and segregation also suggests that double-branched MTOCs with two centrin foci have committed to undergoing a new round of mitosis and that the duplication of cytoplasmic extensions represents the first identifiable step in MTOC duplication. In line with this hypothesis, the most common MTOC states in schizonts prior to segmentation are single MTOCs with 2 cytoplasmic extensions (26% of MTOCs in cells with 2–5 nuclei, 52% in cells with 6–12 nuclei) which have committed to the next round of mitosis and mitotic MTOCs with one extension each (42% of MTOCs in cells with 2–5 nuclei, 36% in cells with 6–12 nuclei) which are finishing a round of mitosis. Interestingly, 8% of MTOCs observed in the 2–5 nuclei stage and 6% in the 6–12 nuclei stage were mitotic and double-branched, suggesting the duplication of cytoplasmic extensions, and commitment to the next round of mitosis, can happen before karyokinesis is completed (Supplementary Figure 2b & 3).

MTOCs reach semi-synchrony at the beginning of segmentation as defined by the first appearance of a basal complex by NHS ester. At this point, rather than seeing a variety of MTOC states and branch numbers, virtually all MTOCs in the same cell share the same mitotic state and branch number. Most MTOCs appear as mitotic MTOCs with a single cytoplasmic extension during early segmentation and then appear as single MTOCs with a single extension during mid-segmentation (Supplementary Figure 2b). By the time segmentation is completed, the MTOC is no longer visible by NHS ester, suggesting that the nuclear MTOC may disassemble after all mitotic events are finished (Supplementary Figure 2b and Figure 2b). Of 159 nuclei imaged in 6 C1-arrested schizonts, none showed the presence of an MTOC.

#### The apical polar rings, Golgi, and rhoptries are all segregated with the MTOC.

Given the cytoplasmic coordination of mitotic events and the physical tethering of the nucleus to the PPM throughout schizogony, we investigated whether we could observe any coordination extending to the apical organelles and other structures known to be present near the MTOC at these stages. We observed close association between the cytoplasmic extensions of the MTOC and the rhoptries, Golgi, basal complex, and APR.

The number of basal complex structures closely matched the number of MTOC cytoplasmic extensions throughout schizogony. No basal complex structures were observed in MTOCs without cytoplasmic extensions. Of 82 single-branched MTOCs imaged, 81 (99%) showed a single basal complex structure. Of 37 double-branched MTOCs imaged, 33 (89%) showed two basal complex structures. As we observed when imaging centrin foci, the few cases when there is a mismatch between the number of cytoplasmic extensions and basal complex structures mostly occur in double-branched MTOCs. However, in this case, the mismatch cannot be attributed to resolution as CINCH staining of the basal complex remains punctate until early segmentation (Figure 4b). Thus, this mismatch between number of cytoplasmic extensions and basal complex structures most likely reflect a transition state. This suggests that basal complex division, when visualized by breaks in CINCH staining, is less simultaneous with MTOC branch duplication than centrin focus duplication.

Rhoptry biogenesis, discussed further below, was also closely tied to the number and position of the MTOC cytoplasmic extensions. This association of one rhoptry per branch, however, is broken at the start of segmentation, when 205 of 211 imaged MTOCs (97%) had a rhoptry pair per MTOC branch. Further suggestive of MTOC-rhoptry interaction is the fact that the rhoptries were positioned immediately next to the MTOC cytoplasmic extensions for as long as these were visible by NHS ester. While we had no APR protein marker, the cytoplasmic extensions always ended in an NHS ester-dense focus at the plasma membrane. At the beginning of segmentation, this focus obtained a morphology suggestive of the APR based on its ring shape, position, and size. As described above, a small percentage of parasites commit to the next round of mitosis before they finish segregating their genetic material. In these parasites, MTOC-associated organelles continued to match the number of cytoplasmic extensions. This gave rise to nuclei that were anchored to four basal complexes, four single rhoptries, and four APR densities in close proximity (Supplementary Figure 3). This matches previous observations by three-dimensional electron microscopy that a single nucleus can have 4 sets of apical buds^16^.

We also characterized the distribution of the Golgi and its association with the MTOC. The Golgi was visualised using an antibody to ER lumen protein retaining receptor 2 (ERD2), a cis-Golgi marker expressed throughout intraerythrocytic development^24^ (Supplementary Figure 4). Small regions of Golgi were visible at all development stages. In ring-stage parasites and mononucleated trophozoites, one or two Golgi foci were observed near the nucleus but had no MTOC association. In all 5 imaged parasites with less than 2 nuclei and lacking MTOC cytoplasmic extensions, no Golgi was observed near the MTOC. In parasites that had undergone the first round of mitosis and had developed MTOC cytoplasmic extensions, the Golgi was proximal to these extensions (Supplementary Figure 2b and Supplementary Figure 4) and closely matched their number and presence. Specifically, 21 of 22 imaged parasites with visible cytoplasmic extensions had Golgi staining associated with each of their MTOCs (Supplementary Figure 2b). While Golgi-MTOC association coincides with the appearance of the cytoplasmic extensions, the Golgi is able to remain at the apical end of the parasite after these tethers and the MTOC are no longer visible by NHS ester. In C1-arrested schizonts, each merozoite has a single Golgi that remains at the apical end of the parasite, typically between the rhoptry bulb and the nucleus (Supplementary Figure 4).

In contrast, we did not observe an MTOC association in the distribution of the endoplasmic reticulum (ER) (Supplementary Figure 5) within the parasite. The ER was visualised using an antibody to Binding Immunoglobulin Protein (BIP), a constitutively expressed ER lumen protein^25^. As expected, ER was detected at all stages of intraerythrocytic development (Supplementary Figure 5). In ring-stage and mononucleated trophozoite-stage parasites, the ER could be seen wrapping around the nucleus and forming recognizable cisternae. In multinucleated parasites, the ER was too dense to observe cisternae, but large regions of the cell were occupied by the ER. Following segmentation in C1-arrested schizonts, the ER was only observed contiguous with the nuclear envelope.

Combining the observations that the MTOC is physically tethered to the PPM by its cytoplasmic extensions and that this anchoring is closely associated with organelles that will define the apical end of the parasite (nuclear pore complexes^10^, Golgi, rhoptries, basal complex, and apical polar rings), we suggest that this tethering by the MTOC cytoplasmic extensions establishes apical-basal polarity in the parasite early in schizogony. Considering that rhoptries are formed from Golgi-derived cargo^26,27^, it is unsurprising to find the Golgi forms part of this apical cluster of organelles throughout schizogony. The confined space between nuclear envelope and PPM that these organelles are packed into, for example, may provide a mechanism for each nucleus to provide rhoptry cargo locally to their own rhoptries rather than to all rhoptries in the cell. The same principle could apply to other apical Golgi-derived organelles. However, it remains unclear what role the MTOC cytoplasmic tethers play in this association, whether any organelles besides the nucleus are physically tethered by these extensions, and how these clusters of organelles remain together during the rapid mitotic events constantly separating sister MTOCs.

#### Characterisation of intranuclear microtubules.

**Figure 3:**
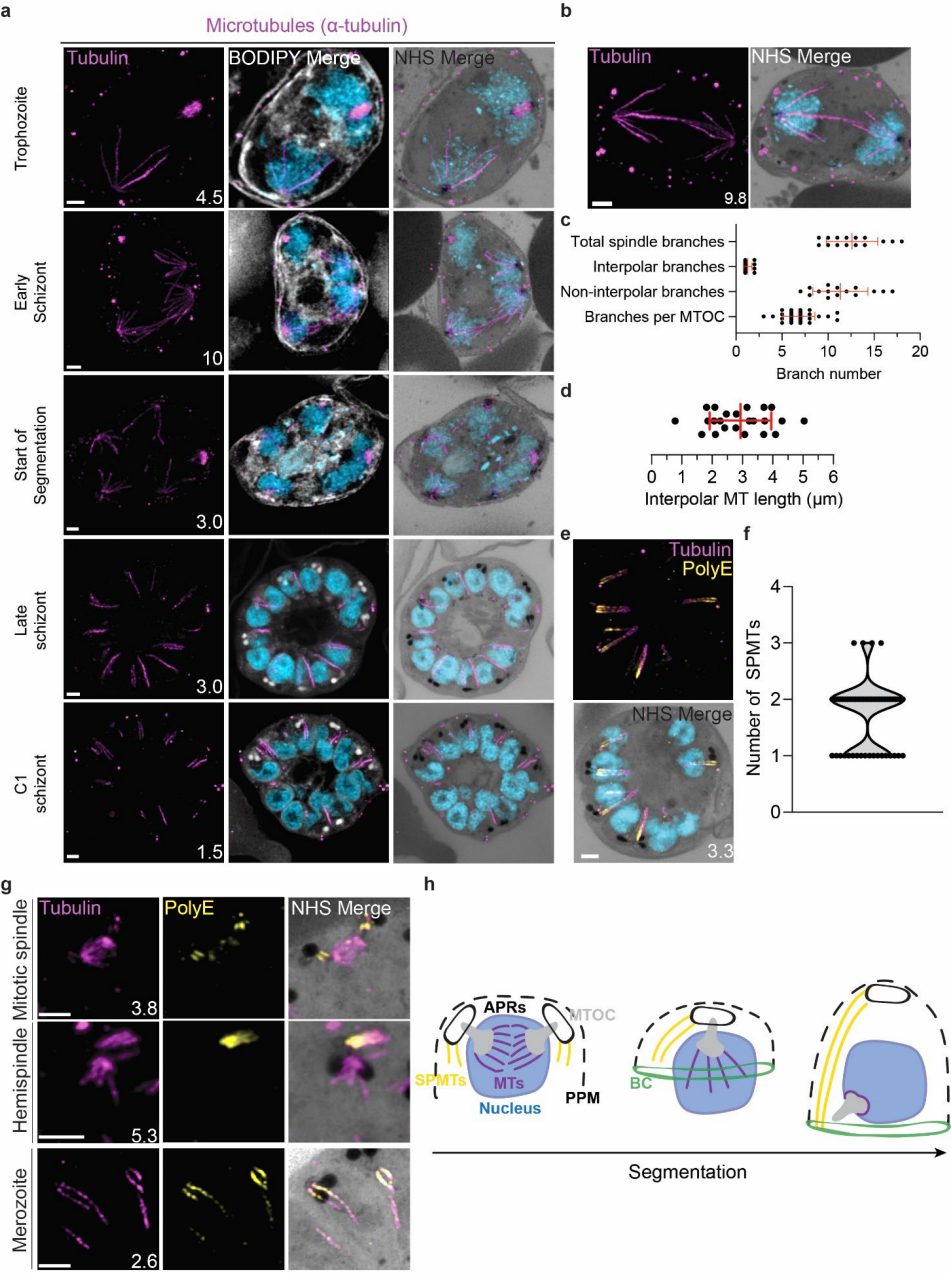
Characterisation of intranuclear and subpellicular microtubules. **(a)** 3D7 parasites were prepared by U-ExM, stained with NHS ester (greyscale), BODIPY TRc (white), SYTOX (cyan) and anti-tubulin (microtubules; magenta) antibodies, and imaged using Airyscan microscopy across the asexual blood stage. **(b)** Nuclei in the process of dividing, with their MTOCs connected by an interpolar spindle. **(c)** The number and type of microtubule branches in interpolar spindles and **(d)** length of interpolar microtubules. **(e)** Subpellicular microtubules (SPMTs) stained with an anti-poly-glutamylation (PolyE; yellow) antibody. **(f)** Quantification of the number of SPMTs per merozoite from C1-treated schizonts. **(g)** SPMT biogenesis throughout segmentation. **(h)** Model for SPMT biogenesis. PPM = parasite plasma membrane, APRs = apical polar rings, BC = basal complex. Images are maximum-intensity projections, number on image = Z-axis thickness of projection in µm. Scale bars = 2 µm.

*P. falciparum* is known to have two classes of microtubules; intranuclear microtubules that partake in mitosis^6,10^, and subpellicular microtubules (SPMTs) that are cytosolic and extend in a single spine from the apical end of merozoites^6,10^. Investigating microtubules with an anti-α-tubulin antibody, we failed to detect microtubules in ring-stage parasites, consistent with previous observations (Supplementary Figure 6)^10^. Intranuclear microtubules were first visible in mononucleated trophozoite-stage parasites and were present until early segmentation stages, with no intranuclear microtubules visible by the end of segmentation (Figure 3a). Intranuclear microtubules arrange into three distinct spindle structures: hemispindles, mitotic spindles, and interpolar spindles. Hemispindles are microtubule structures coming from a single MTOC that retract prior to MTOC duplication. Mitotic spindles appear following MTOC duplication and separate sister chromatids during mitosis. When the two MTOCs migrate away from each other, they remain connected by an elongated microtubule structure called the interpolar spindle (or elongated spindle), which retracts prior to nuclear fission^10,28,29^. It has recently been shown that the interpolar spindle is short-lived relative to the hemispindle and mitotic spindle^10,28^. In this study, we observed 24 interpolar spindles, which allowed us to perform the first detailed characterisation of this spindle type (Figure 3b).

Interpolar spindles have microtubule branches that connect the two distant MTOCs (interpolar microtubules), and microtubule branches that do not connect the MTOCs (non-interpolar microtubules). Each interpolar spindle contained an average of 12.5 (± 2.6 SD) total microtubules, of which 1.3 (± 0.6 SD) were interpolar microtubules and 11.2 (± 2.8 SD) were non-interpolar microtubules (Figure 3c). The average number of non-interpolar branches per MTOC was 5.6, which is similar to the previously reported average number of branches in a hemispindle of 5 to 6^6,10^. This suggests that only the interpolar microtubules retract during the interpolar spindle to hemispindle transition. We measured interpolar microtubules in 3D, adjusting for expansion factor by dividing the measured distance by 4.25, the median expansion factor observed in this study (Supplementary Figure 7a&b; Materials and Methods). Interpolar microtubules ranged from ~1–5 µm, with a mean length of 2.9 µm (± 1.0 µm SD) or 12.47 µm before expansion factor correction (Figure 3d). In all cases, the MTOCs connected by interpolar spindles were anchored to the plasma membrane by their cytoplasmic extensions. The large variability in interpolar microtubule size and the continued tethering of the MTOCs to the PPM suggest that interpolar microtubules push PPM-anchored MTOCs to opposite sides of the cell without causing detachment from the PPM. It is unclear how parasites achieve this sliding effect or how MTOC-associated organelles are able to retain this association while MTOCs are moved large distances.

#### Subpellicular microtubule length and biogenesis.

Subpellicular microtubules are nucleated in the cytoplasm and have long been observed in merozoites^30^. SPMTs have been shown to be stabilised by polyglutamylation^5^ and can be identified specifically using a combination of anti-tubulin and anti-PolyE antibodies (Figure 3e). Using this approach, we characterised 86 SPMTs in 50 merozoites from C1-arrested schizonts. These nascent merozoites had between 1 and 3 SPMTs, with an average of 1.7 (± 0.6 SD) (Figure 3f). Of 50 imaged merozoites, 48 had at least one SPMT that extended >50% of cell length from the apical polar ring to the basal complex. This longest microtubule in a merozoite had an average length of 1.01 µm (± 0.24 µm SD). In merozoites with more than one SPMT, the second and third microtubules were shorter than the first, having an average length of 0.8 µm (± 0.21 µm SD). Given the large variation in SPMT size and observation that, in segmenting schizonts, the basal end of the SPMTs was in contact with the basal complex throughout segmentation, we hypothesise that most SPMTs measured in our C1-treated schizonts had partially depolymerised. *P. falciparum* microtubules are known to rapidly depolymerise during fixation^10,29^. It is unclear, however, why this depolymerization was observed most often in C1-arrested parasites. Thus, we cannot determine whether these shorter microtubules are a by-product of drug-induced arrest or a biologically relevant native state that occurs at the end of segmentation.

Little is known about SPMT biogenesis during the asexual blood stage of *P. falciparum*, but it is currently hypothesized that they are nucleated by the apical polar rings^31,32^, as is the case in *Toxoplasma*^32,33^. Curiously, TgCentrin 2 localizes to the apical polar ring of *Toxoplasma* tachyzoites^34^, but no Centrin 2 has been observed to localize to the apical polar rings of *P. falciparum*. Furthermore, it was recently shown that the SPMTs of *P. falciparum* gametocytes, which lack an APR, are formed at the cytoplasmic extensions of the nuclear MTOC, in the space between the nuclear envelope and PPM^22^. Leveraging our ability to specifically detect SPMTs using PolyE, we investigated the possibility that merozoite SPMTs are also formed at the cytoplasmic extensions and subsequently transferred onto the apical polar ring during segmentation. In schizonts where nuclei are approaching or have completed their final mitosis (~15n), we observed small cytoplasmic microtubules that stained strongly with PolyE appear in the area between the nuclear MTOC and PPM (Figure 3g). However, we did not achieve a resolution that allowed us to distinguish individual APRs or to confidently pinpoint whether the microtubules were nucleated at the APRs or the cytoplasmic extensions. Likely, higher resolution imaging techniques are needed to resolve the site of SPMT nucleation in merozoites.

### Segmentation machinery (inner membrane complex and basal complex)

Following replication of their genetic material during the trophozoite and early schizont stages, parasites partition their nuclei and organelles into ~30 daughter merozoites from the common cytoplasm of a schizont^35^. This form of cytokinesis, called segmentation, takes place in the final hours of schizogony and culminates with the physical separation of each daughter cell and their egress from the host RBC. The inner membrane complex (IMC) is a double lipid bilayer formed from flattened vesicles that scaffolds the process of segmentation as well as anchors many proteins important for parasite shape and motility^36^.

#### The IMC cannot be distinguished from the plasma membrane by U-ExM

The IMC forms *de novo* during segmentation starting at the apical end of the parasite, where the MTOC is anchored to the plasma membrane (Supplementary Figure 8a)^36^. This can be observed using the IMC-anchored protein Glideosome associated protein 45 (GAP45), which bridges the IMC and plasma membrane, as well as using BODIPY TRc, which shows increased membrane staining in the area overlapping GAP45^37^ (Supplementary figure 8a). As segmentation progresses, the IMC expands around the nucleus and associated organelles until it envelops the daughter cell, leaving an opening at the apical end, where the apical polar ring is located, and the basal end, where the basal complex resides (Supplementary Figure 8a). While the pellicle was easily visualized as a whole, we were unable to distinguish the IMC membranes from the PPM (Supplementary Figure 8b&c). We stained parasites using the plasma membrane marker MSP1 and two different IMC markers: GAP45, which lies between the IMC and PPM, and IMC1g, which is attached to the cytoplasmic face of the IMC^38,39^. In both cases we were unable to resolve the IMC marker from MSP1 (Supplementary Figure 8b&c).

#### Basal complex dynamics throughout segmentation

**Figure 4:**
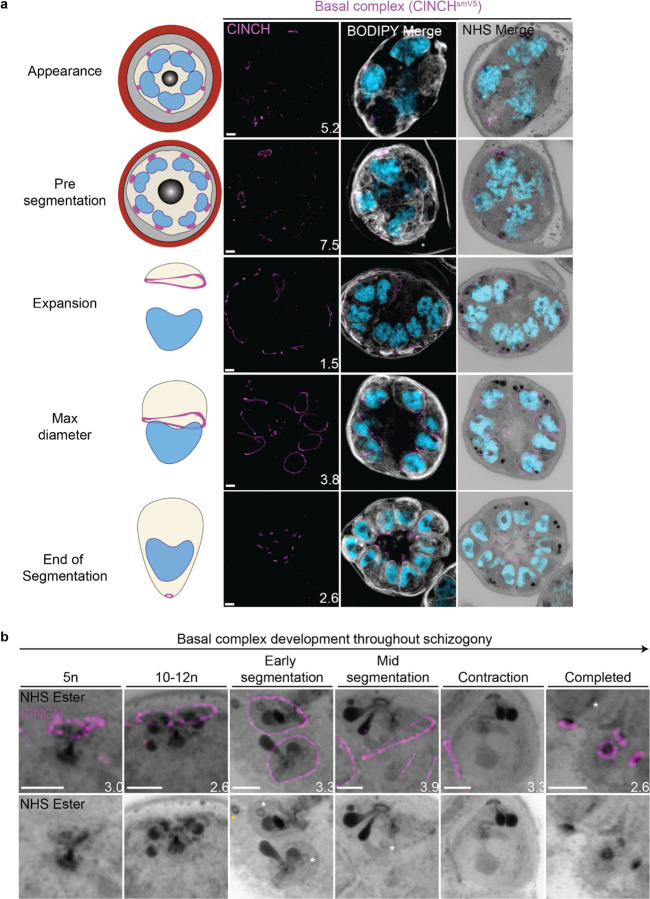
Basal complex biogenesis and development throughout segmentation. **(a)** Parasites expressing an smV5-tagged copy of the basal complex marker CINCH were prepared by U-ExM, stained with NHS ester (greyscale), BODIPY TRc (white), SYTOX (cyan) and anti-V5 (basal complex; magenta) antibodies and imaged using Airyscan microscopy across segmentation. **(b)** Basal complex development during schizogony. The basal complex is formed around the PPM anchor of the MTOC. In nuclei whose MTOC has two cytoplasmic extensions, and will therefore undergo mitosis, the basal complex rings are duplicated. From early segmentation, the basal complex acquires a stable, expanding ring form. Cytostomes that will form part of merozoites are marked with a white asterisk, while those outside merozoites are marked with a yellow asterisk. Images are maximum-intensity projections, number on image = Z-axis thickness of projection in µm. Scale bars = 2 µm.

The basal complex is an essential ring structure located at the basal end of the IMC^40^. It is hypothesized to act as a contractile ring that guides IMC biogenesis and mediates abscission of newly formed merozoites by separating the IMC and plasma membrane from the residual body. We used parasites where PfCINCH, a basal complex marker, was tagged with an smV5 tag to follow basal complex development throughout schizogony (Figure 4)^41^. CINCH is first visible at early schizogony (3–5 nuclei stage) as a small ring-like structure surrounding an NHS ester-dense focus on the plasma membrane that is tethered to the MTOC (Figure 4). Of 55 early schizont MTOCs imaged, 44 (80%) had matching numbers of MTOC cytoplasmic extensions and basal complex structures. This suggests that as MTOCs divide, they each inherit a CINCH ring that has been split by the duplication of the cytoplasmic tethers (Figure 4b). During the rapid nuclear divisions of schizogony, 77% of the CINCH structures of mitotic MTOCs show a break in the ring. This break faces a sister basal complex with its own cytoplasmic extension (Figure 4b and Supplementary Figure 3). So, when we see duplication of the MTOC’s cytoplasmic extensions, the basal complex ring “breaks” into two semicircles which re-seal to form their own ring prior to the next branch duplication (Figure 4b).

Once segmentation begins and the MTOC’s cytoplasmic extensions stop duplicating, CINCH forms a *bona fide* ring with smooth borders (Figure 4). At this point, nuclei reach a point of semi-synchronicity. All 64 imaged MTOCs in early segmentation parasites had duplicated MTOCs forming a mitotic spindle and each MTOC had a single uninterrupted basal complex ring (Figure 4b). This event marks the last nuclear division the parasite will undergo. Thus, we cease to observe events where a single nucleus is attached to four basal complexes as MTOCs have ceased committing to future rounds of mitosis. By the time the basal complex reaches its maximum diameter, all nuclear divisions have been completed, each nucleus has a single MTOC and basal complex, and no mitotic spindles are visible (Figure 4). After this point, the basal complex contracts and moves in the basal direction away from the apical end. By the time segmentation is completed, the basal complex is an NHS ester dense ring that is smaller than the apical polar ring.

#### NHS ester as a basal complex marker

While the basal complex stains brightly with NHS ester at the end of segmentation (Figure 4), this staining is not consistent throughout schizogony. Since NHS ester is an indicator of protein density, changes in basal complex staining could indicate a relative change in protein abundance at the basal complex. NHS-ester staining of the basal complex is not visible or is very faint during early schizogony. Once the basal complex attains its *bona fide* ring form during early segmentation, it stains reliably, though faintly, with NHS ester. This staining intensifies after the basal complex begins to contract. This denser staining could be due to recruitment of more basal complex proteins at the midpoint of schizogony, an increase in protein density as the ring area decreases during contraction, or both. Once the parasites finish segmentation, the basal complex is at its brightest (Figure 4b). While NHS ester staining correlates with CINCH, it does not perfectly overlap with it. CINCH consistently appears as a larger ring with a slight basal shift relative to NHS ester after the basal complex reaches maximum diameter, an effect most visible at the end of segmentation (Figure 4b). Since this shift is consistent with parasite anatomy regardless of parasite orientation, it suggests it is not an imaging artifact. There is no primary antibody against CINCH at this point, and so it is not possible to determine whether the lack of overlap with NHS ester is due to distance between the smV5 tag and the main protein density of CINCH (CINCH is 230kDa). It is also possible that this difference in localization reflects basal complex architecture similar to that previously observed in *Toxoplasma gondii*, where the basal complex consists of multiple concentric rings^34,42,43 44^.

### Mitochondrion and apicoplast

The apicoplast and mitochondrion undergo pronounced morphological changes during the *P. falciparum* blood-stage lifecycle ^45,46^. Both are long, and often branching, organelles whose complex three-dimensional morphologies have only been robustly studied using electron microscopy-based techniques^16^.

#### Looped regions of the mitochondrion display low membrane potential.

To visualize the mitochondria, we stained live parasites using Mitotracker Orange CMTMRos prior to fixation and expansion (Supplementary Figure 9a). Mitotracker Orange CMTMRos accumulates in live mitochondria, driven electrophoretically by membrane potential, and is retained after fixation^47,48^. When imaged at high resolution, Mitotracker can be used to observe individual cristae in the mitochondria of mammalian cells^49^. *Plasmodium* cristae morphology is different from that found in mammalian mitochondria; cristae are thought to be bulbous or tubular rather than lamellar and are present in gametocytes but absent from asexual blood-stage parasites^50,51^. To our surprise, rather than showing continuous staining of the mitochondria, Mitotracker staining of our expanded parasites revealed alternating regions of bright and dim staining that formed Mitotracker-enriched pockets (Supplementary Figure 9b). These clustered areas of Mitotracker staining were highly heterogeneous in size and pattern and seemed to indicate that regions of high membrane potential are confined to specific regions of the organelle. Small staining discontinuities like these are commonly observed in mammalian cells when using Mitotracker dyes due to the heterogeneity of membrane potential from cristae to cristae as well as due to fixation artifacts. While our observed Mitotracker-enriched pockets could be a fixation artifact or be a product of local membrane depolarization, we cannot discard the possibility that regions of brighter staining represent Mitotracker accumulation inside irregular compartments of high membrane potential within the mitochondrion.

In addition to these small staining discontinuities, we observed large gaps in Mitotracker staining within parasites at all stages of development. This included pre-segmentation parasites, where we would expect a single continuous mitochondrion to be present (Supplementary Figure 9a). To our knowledge, no membrane potential discontinuities or fixation artifacts of this size have been reported in mammalian cells. So, as a secondary way to visualise the mitochondria and better characterize these staining discontinuities, we generated a transgenic cell line with the putative ATP synthase F0 subunit-d (ATPd, Pf3D7_0311800) tagged with a spaghetti monster HA tag^52^ (Supplementary Figure 10). ATPd is a membrane-embedded proton channel that had not previously been localized to the mitochondria in *P. falciparum* but was identified as a mitochondrial protein in a recent proteomics study^51,53^. Furthermore, its *Toxoplasma* homologue has been shown to localize to the mitochondria^54,55^. We confirmed that ATP synthase subunit F0 localizes to *P. falciparum* mitochondria, as it largely co-localized with Mitotracker staining, forming a border around it due to its membrane association (Supplementary Figure 9a). ATPd, like Mitotracker, had a heterogenous distribution throughout the mitochondria, but it did not show the same large gaps in staining. ATPd allowed us to better visualize regions of the mitochondria that appeared to fold onto themselves and fuse with each other, as has been previously described^45^. Thus, Mitotracker and ATPd are both useful but imperfect markers for the mitochondria, with neither of them showing a continuous, even distribution throughout the organelle.

**Figure 5:**
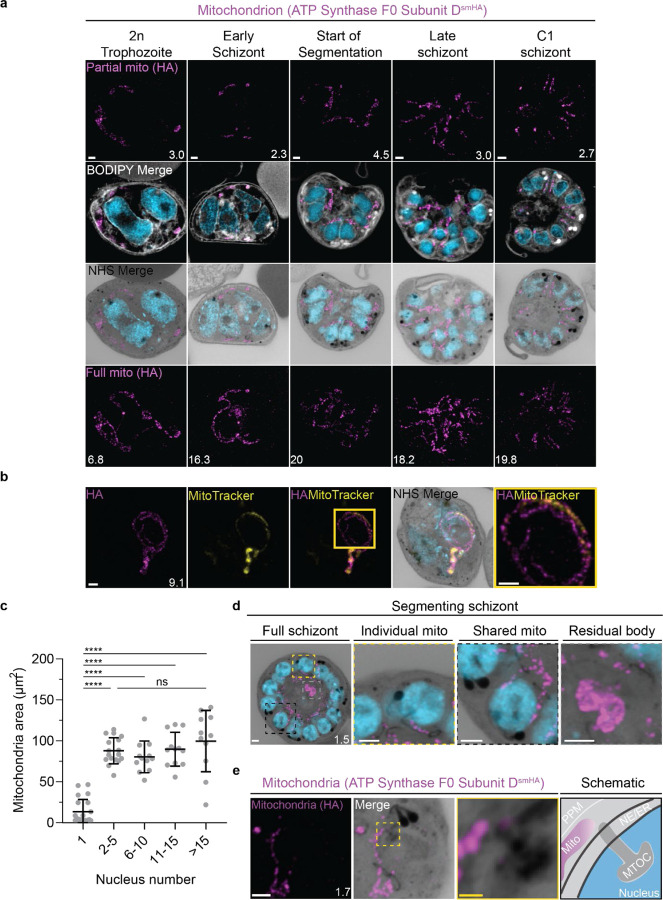
Growth and fission of the mitochondrion. **(a)** Parasites with an smHA-tagged copy of the ATP Synthase F0 Subunit D (ATPd, Pf3D7_0311800) as a mitochondrial marker were prepared by U-ExM, stained with NHS ester (greyscale), BODIPY TRc (white), SYTOX (cyan) and anti-HA (mitochondrion; magenta) antibodies and imaged using Airyscan microscopy across the asexual blood stage. Maximum intensity projections of both a subsection of the cell (partial mito) and the full cell (full mito) are shown. **(b)** ATPd staining was compared against Mitotracker orange CMTMRos (yellow), which showed discontinuous staining in looped regions. **(c)** Area of the mitochondrion was quantified for parasites of varying nucleus number. 73 cells were counted across 4 biological replicates. **** = p<0.001, ns = p>0.05 by one-way ANOVA, error bars = SD. **(d)** Schizont with mitochondria that have undergone fission (yellow zoom), mitochondria that are shared between two nascent merozoites (black zoom), and mitochondria left outside merozoites in the forming residual body (grey). **(e)** During fission, mitochondria associate with the cytoplasmic extension of the MTOC. Images are maximum-intensity projections, number on image = Z-axis thickness of projection in µm. White scale bars = 2 µm, yellow scale bars = 500 nm.

Curiously, 25 of 26 imaged parasites showed Mitotracker discontinuities specifically in regions where the ATPd signal formed looped structures (Figure 5b). These structures were defined as areas where the mitochondria showed a turn or fold of ~180°. Of the 41 looped regions identified, 75% lacked Mitotracker staining. This suggests that mitochondria looped regions in *P. falciparum* have some degree of depolarization that prevents Mitotracker accumulation or that Mitotracker initially accumulates in these regions but is not bound and retained. The biological significance of these areas, if any, is currently unclear.

#### Growth of the apicoplast and mitochondrion

**Figure 6:**
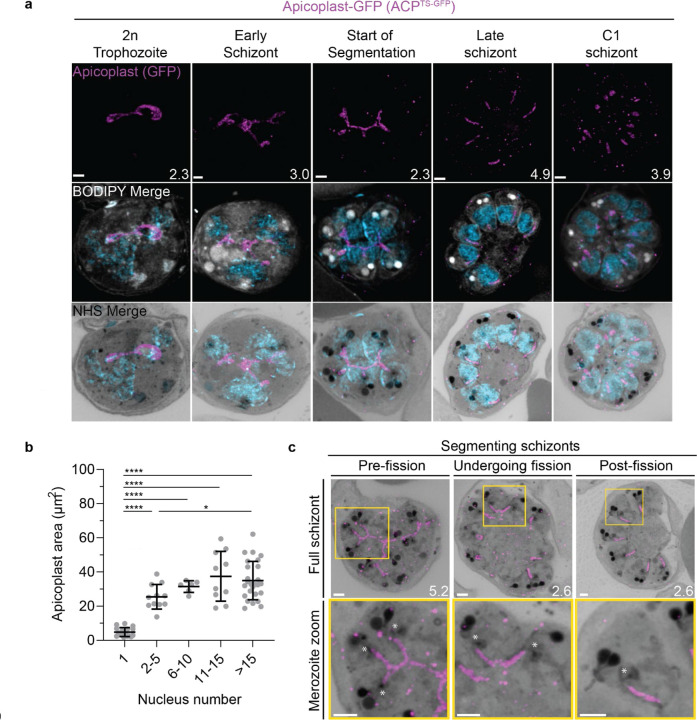
Growth and fission of the apicoplast. **(a)** Parasites expressing GFP-conjugated to the apicoplast transit signal of ACP (ACP^Ts-GFP^) were prepared by U-ExM, stained with NHS ester (greyscale), BODIPY TRc (white), SYTOX (cyan) and anti-GFP (apicoplast) (magenta) antibodies and using Airyscan microscopy across the asexual blood stage. **(b)** Area of the apicoplast was quantified for parasites of varying nucleus number. 70 cells were counted across 3 biological replicates. **** = p<0.001, * = p<0.05 by one-way ANOVA, error bars = SD. **(c)** Representative images of the different stages of apicoplast fission. Images are maximum-intensity projections, number on image = Z-axis thickness of projection in µm. Asterisks represent MTOCs. Scale bars = 2 µm.

To visualize the apicoplast, we utilized a previously established cell line that expresses GFP fused to the apicoplast transit peptide of acyl carrier protein (ACP)^56^, which we will refer to as Apicoplast-GFP. This marker allowed for a relatively even and continuous staining of the organelle. We quantified mitochondrion and apicoplast signal area using ATPd-smHA and Apicoplast-GFP respectively as a proxy measurement of size (Figure 5c&6b). Tracking this in parallel to parasite nucleus number allowed us to determine whether the growth of these organelles occurred progressively with simultaneous rounds of mitosis and nuclear division. In mononucleated ring and trophozoite parasites, both the mitochondria and the apicoplast are relatively small, having an average area of 13.39 µm^2^ (± 15.1 µm^2^ SD) and 4.81 µm^2^ (± 2.62 µm^2^ SD) respectively in expanded parasites (Supplementary Figure 6). As expected from live cell observations^45^, both organelles show significant growth and spread throughout the cell in multinucleated parasites, adopting an elongated and branching morphology (Figure 5&6). Mitochondria grow almost exclusively during the first two rounds of nuclear replication, achieving an average size of 87.9 µm^2^ (± 15.9 µm^2^ SD) at the 2–5 nuclei stage. This size remains relatively constant until segmentation, with the average mitochondria size right before the start of fission being 99.6 µm^2^ (± 37.4 µm^2^ SD) (Figure 5c). In contrast, the apicoplast continues to grow past the 2–5 nuclei stage, having an average size of 25.5 µm^2^ (± 7.21 µm^2^ SD) at the 2–5 nuclei stage and 34.97 µm^2^ (± 11.23 µm^2^ SD) in cells with >15 nuclei (Figure 6b). These data suggest that the mitochondrion and apicoplast do not grow simultaneously with or as a response to nuclear replication during schizogony. Rather, both organelles show the largest increase in size during the 1 to 2 nuclei transition and either plateau in size, in the case of the mitochondria, or enter a second phase of slower growth that ends shortly before segmentation, in the case of the apicoplast.

#### Fission of the mitochondrion and apicoplast

*P. falciparum* has a single, large, branching, mitochondrion and apicoplast throughout most of the asexual blood stage^45,46,57^. During segmentation, however, these organelles undergo fission such that each merozoite inherits an individual apicoplast and mitochondrion^16,45^. While it has been shown that apicoplast fission occurs before mitochondrial fission, it is unclear how fission occurs^16^. A recent review^57^ posed three possible mechanisms: synchronous fission where the organelle simultaneously divides into all daughter parasites at once, outside-in fission where fission occurs at the ends of the organelle, or branching point fission where a first fission event divides the organelle into larger segments and a subsequent fission event leaves each merozoite with an individual organelle^57^. It also remains unclear how accurate segregation into daughter cells is monitored. In *T. gondii*, the apicoplast associates with the centrosomes prior to undergoing fission. A similar association between apicoplasts and MTOCs has been proposed in *Plasmodium* but still lacks evidence due to the difficulty of observing the *Plasmodium* MTOC in live cells^45^. The mitochondrion is not thought to associate with the MTOC in *Toxoplasma* or *Plasmodium* and its mechanism for ensuring accurate segregation remains unknown.

In the process of imaging the mitochondria and apicoplasts of segmenting parasites, we observed a transient MTOC association prior to and during fission in both organelles. Just before the start of segmentation, there is little association between the MTOC and apicoplast. Of 5 parasites imaged that had >10 nuclei but had not yet started segmentation, 3 had no contact points between the MTOC and apicoplast branches and the other 2 had <4 contact points. Once segmentation starts, all apicoplast branches contact an MTOC each and remain in contact with the MTOC until the end of apicoplast fission and MTOC degradation (Figure 6c). In 11 imaged segmenting schizonts, all MTOCs showed contact with an apicoplast branch each. This association starts in early segmentation, when all MTOCs are connected by a single branched apicoplast. By the time the basal complex reaches maximum diameter and begins contraction, we observe 7 parasites where all branches have completed fission and 2 parasites that had at least one or more apicoplast segments still connecting multiple merozoites (Figure 6c). Mitochondria fission follows a very similar pattern, but later in parasite development. Prior to the basal complex reaching maximum diameter, we observe no significant connection between the MTOC and mitochondria. When the basal complex entered its contraction phase, we observed 9 parasites where all MTOCs were in contact with one branch of the mitochondria each (Figure 5 d&e). Only one of these 9 parasites had an intact pre-fission mitochondrion, while the other 8 had undergone at least one fission event. Matching previous descriptions of mitochondria and apicoplast segmentation, C1-arrested schizonts having completed segmentation show elongated mitochondria (Figure 5a) and small, rounded apicoplasts (Figure 6a).

Both mitochondria and apicoplast fission showed neighbouring nascent merozoites that shared a single branch of mitochondria or apicoplast passing through both of their basal complexes while others had an individual mitochondrion or apicoplast which had already separated from the rest (Figure 5d&6c). This suggests that fission does not occur synchronously (Figure 5d) and supports the model of branching point fission. In other words, parasites seem to undergo a primary fission event that leaves only some merozoites sharing stretches of the organelles and then a subsequent fission event leaves each merozoite with an individual apicoplast and mitochondrion (Figure 5e). Unfortunately, BODIPY TRc does not distinctly stain the membranes of the mitochondria and apicoplast. So, it is not possible for us to determine whether the observed breaks in staining of our chosen organelle markers truly indicate a complete fission of the mitochondria or apicoplast membranes. Thus, while suggestive of branching point fission, our data is not sufficient to conclusively determine the sequence of fission events in these organelles.

#### Characterisation of residual body mitochondria

At the completion of segmentation, the parasite forms a structure known as the residual body, which contains parasite material, such as the hemozoin crystal, that was not incorporated into merozoites during segmentation^16^. The residual body is poorly understood in *Plasmodium*, but in *Toxoplasma* it has been shown that a significant amount of the mitochondria, and not the apicoplast, is left behind in the residual body following segmentation^58^.

There is no well-characterized marker of the residual body in *Plasmodium*. So, for this study, we defined the residual body as any area within the parasitophorous vacuole membrane but visibly external to any merozoite in a C1-arrested schizont as determined by BODIPY TRc staining. We imaged 35 C1-arrested schizonts and observed that 54% had mitochondrial staining inside the residual body (Figure 5d and Supplementary Figure 9c). To determine the proportion of total mitochondria that gets included in the residual body, we quantified the fluorescence of both mitochondria in the residual body and mitochondria in merozoites. Of the 19 parasites that showed mitochondria staining inside the residual body, the amount of material ranged from 1% to 12% of the total mitochondrial staining in the parasite (Supplementary figure 9d). On average, the residual body had approximately 1.5x more mitochondrial staining than the average merozoite (Supplementary Figure 9d). No significant apicoplast staining was ever observed in the residual body, similarly to what has been reported for *Toxoplasma*^58^.

### Cytostomes

During its intraerythrocytic development, *P. falciparum* engulfs host cell cytoplasm from which it catabolizes haemoglobin as a source of amino acids^59^. The parasite is separated from its host cell by the parasitophorous vacuole, and therefore the uptake of host-cell cytosol requires invagination of both the PPM and PVM. The cytostome coordinates this endocytic process and is comprised of two key regions: a protein-dense collar region, which forms the pore through which membrane invagination will occur, and the membranous bulb region, which contains the RBC-derived cargo^60,61^.

#### NHS ester staining reveals pore-like structures at the parasite plasma membrane.

**Figure 7:**
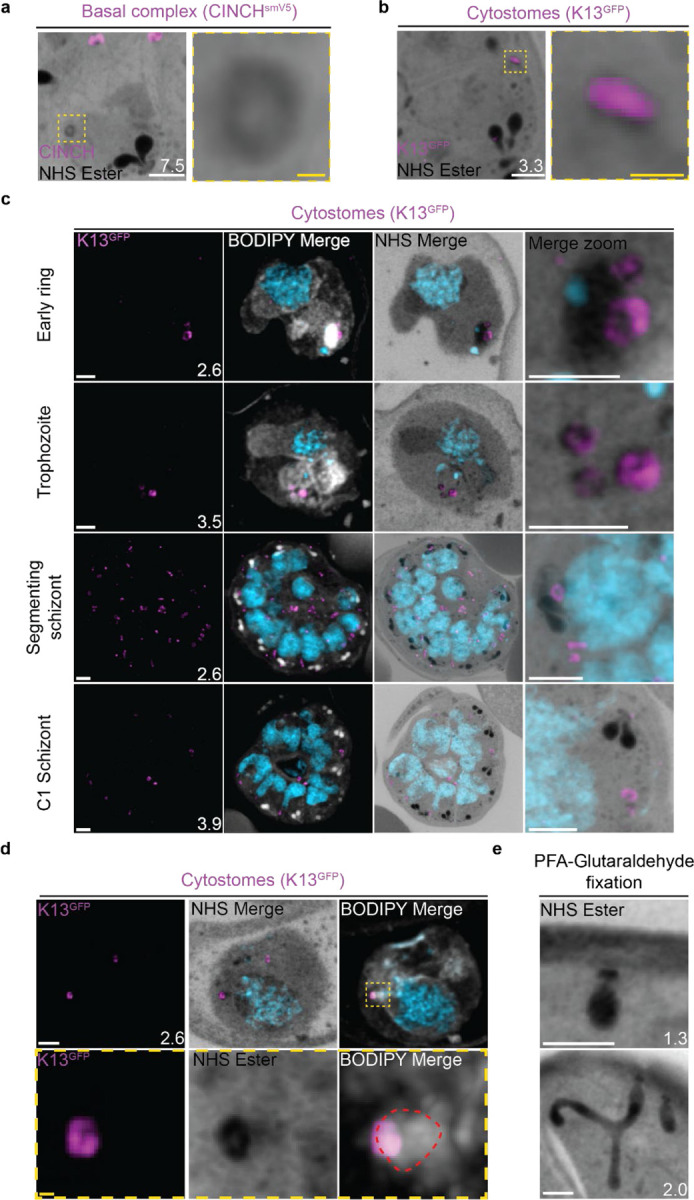
Cytostomes are observable by U-ExM throughout the asexual blood stage of the lifecycle. **(a)** Parasites expressing an smV5-tagged copy of the basal complex marker CINCH and stained anti-V5, show NHS ester dense rings that are negative for this basal complex marker. **(b)** Parasites where the cytostome marker Kelch13 was conjugated to GFP (K13-GFP), showed overlap between K13 and this non-basal complex structure when stained against GFP. **(c)** K13-GFP parasites were prepared by U-ExM, stained with NHS ester (greyscale), BODIPY TRc (white), SYTOX (cyan) and anti-GFP (Cytostome) (magenta) antibodies and imaged using Airyscan microscopy across the asexual blood stage. **(d)** Paraformaldehyde (PFA) fixed parasites and zoom into region marked by yellow dotted line. Red dotted line marks example of a vesicle attached to the cytostome collar occasionally visible by NHS ester or BODIPY. **(e)** In PFA-glutaraldehyde fixed samples, cytostome collars were attached to protein-dense bulb regions. Images are maximum-intensity projections, number on image = Z-axis thickness of projection in µm. White scale bars = 2 µm. Yellow scale bars = 500 nm.

Prior to this study, cytostomes were not immediately obvious by NHS ester staining given the large number of features that were visible using this stain but pending validation. While observing the basal complex of segmenting schizonts (Figure 4), we noticed that merozoites contained a second NHS-ester-dense ring (Figure 4b and Figure 7a). The size and position of this NHS ester ring matched that of an endocytic micropore recently identified in *Toxoplasma* tachyzoites^62^. In that study, the micropore was identified using Kelch13 (K13) as a marker ^62^. To investigate whether this NHS-ester-dense ring was indeed the K13 micropore, we evaluated a parasite strain where the endogenous K13 was fused to GFP^63^. Investigation using this parasite line revealed that the NHS-ester-dense ring also stained with K13, suggesting that this structure is an endocytic micropore (Figure 7b). Furthermore, K13-stained micropores were detected at all stages of the parasite lifecycle (Figure 7c). Ring-stage parasites typically contained one or two micropores, which increased in number during the trophozoite stage and schizogony (Figure 7c).

Single cytostomes appear in the area containing the IMC near the apical organelles at the same time as the basal complex forms a complete ring. Cytostomes remain within the IMC area but change positions within the nascent merozoite as segmentation progresses (Figure 4b, white asterisks). The majority of merozoites in C1-arrested schizonts contained a single cytostome. This suggests that cytostomes are incorporated into the IMC of merozoites and inherited early in segmentation. Clusters of cytostomes that had not been incorporated into merozoites during segmentation were observed either adjacent to nascent merozoites or as part of the residual body (Supplementary Figure 11 and Figure 4b, yellow asterisk). It is currently unclear whether there are any functional differences between the cytostomes that are incorporated into merozoites and those that are left behind.

#### The observed micropore is the cytostome collar.

K13 should localize to the collar of cytostomes when the parasite is performing endocytosis^64^. While many K13-positive micropores were present at the lifecycle stages when endocytosis is expected (Figure 7c), they were not attached to cargo-containing, protein-dense bulbs indicative of active endocytosis^61^. Occasionally, K13-micropores were attached to membranous structures visible either by BODIPY TRc staining (Figure 7d) or by a decrease in NHS ester staining (Supplementary Figure 11), but these did not match the density or complexity suggested by electron microscopy studies of the same structures. Fixation using 4% v/v paraformaldehyde (PFA) is known to result in the permeabilization of the RBC membrane and loss of its cytoplasmic contents^65^. Topologically, the cytostome is contiguous with the RBC cytoplasm and so we hypothesised that PFA fixation was resulting in the loss of cytostomal contents and obscuring of the bulb. PFA-glutaraldehyde fixation has been shown to better preserve the RBC cytoplasm^65^. So, we prepared samples using this fixative and observed large protein-dense cytostome bulbs (Figure 7e). Collectively, this data supports the hypothesis that these NHS-ester-dense rings are indeed cytostomes and that endocytosis can be studied using U-ExM, but PFA-glutaraldehyde fixation is required to maintain cytostome bulb integrity.

#### Non-canonical cytostome collar morphologies

We noticed a number of different cytostome morphologies and organizational patterns (Supplementary Figure 11). Cytostomes frequently clustered together and did not appear randomly distributed across the PPM (Figure 7c). Some cytostomes would form what appeared to be higher order structures where two or three distinct cytostomal collars appeared to be stacked end-on-end (Supplementary Figure 11). Cytostomes have a relatively well defined and consistent size^64,66^, but occasionally we observed very large cytostomal collars that were approximately twice the diameter of other cytostome collars in the same cell (Supplementary Figure 11). It is unclear what the function of these higher order structures or large cytostomes is, if they represent biogenesis transition states, or indeed if they’re performing some specialized endocytosis.

### The rhoptries

To invade host red blood cells, merozoites secrete proteins from specialized secretory organelles known as the rhoptries and micronemes. While both the rhoptries and micronemes are well studied in the context of *Plasmodium* biology, neither have been investigated in detail using expansion microscopy in *Plasmodium*. We previously showed that fully formed rhoptries can be observed by NHS ester staining alone^6^, but did not investigate their biogenesis.

#### Rhoptries can be observed from early in their biogenesis using NHS ester staining.

Rhoptries consist of a neck and bulb region, with the tip of the neck being loaded into the apical polar rings of merozoites. Despite both being formed from Golgi-derived cargo, the neck and bulb regions have distinct proteomes^26^. We first tracked rhoptry bulb biogenesis across schizogony using antibodies directed against the rhoptry bulb marker rhoptry associated protein 1 (RAP1).

**Figure 8:**
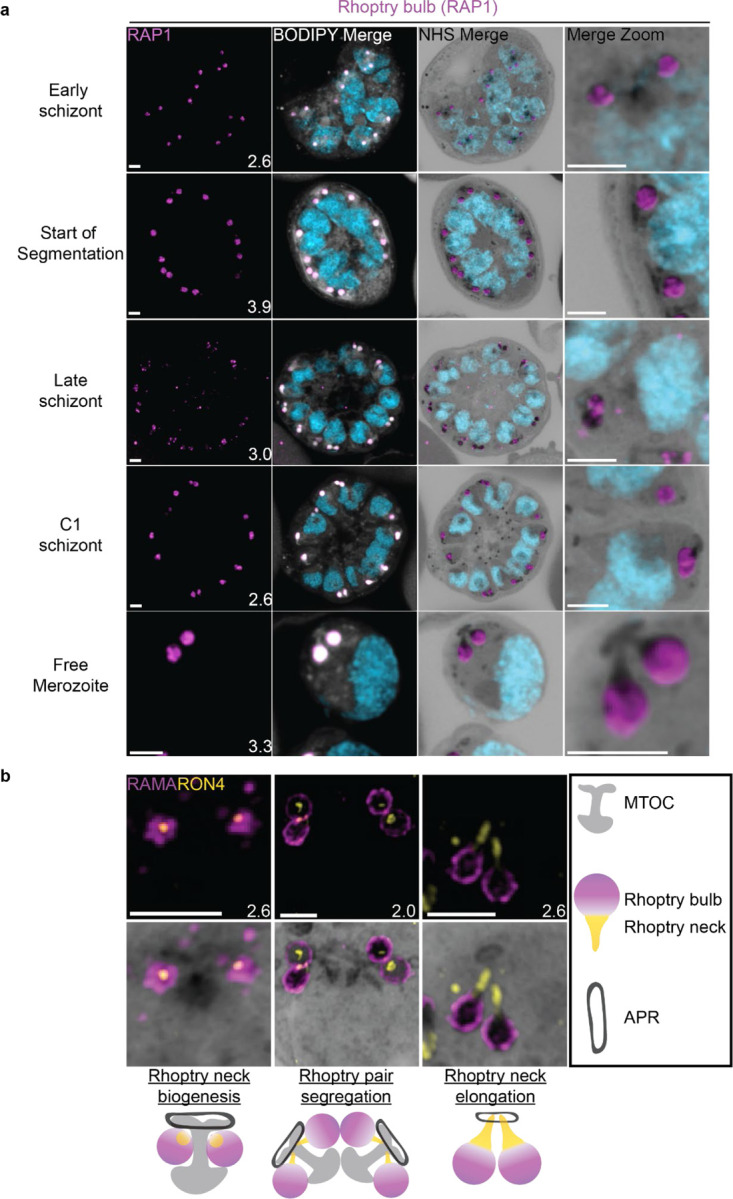
Rhoptries undergo biogenesis near the MTOC and are segregated during nuclear division. **(a)** 3D7 parasites were prepared by U-ExM, stained with NHS ester (greyscale), BODIPY TRc (white), SYTOX (cyan) and anti-RAP1 (rhoptry bulb; magenta) antibodies and imaged using Airyscan microscopy throughout schizogony. **(b)** 3D7 parasites were stained with NHS Ester (greyscale) along with antibodies against RAMA (rhoptry bulb; magenta) and RON4 (rhoptry neck; yellow) to assess rhoptry enck biogenesis. We observed that the rhoptry neck begins as a single focus inside each rhoptry. Rhoptries then get duplicated and segregated alongside the MTOC. During the final mitosis, the rhoptry neck begins to elongate and the rhoptries separate from MTOC. Images are maximum-intensity projections, number on image = Z-axis thickness of projection in µm . Scale bars = 2 µm.

Nascent rhoptries were detected early in schizogony, with RAP1 foci appearing adjacent to all MTOC cytoplasmic extensions from parasites with 6–10 nuclei in a one-to-one ratio, as described above (Figure 8a and Supplementary Figure 12b). These foci co-localized with NHS ester densities of the same size and round shape, no elongated neck-like structures were visible by NHS ester (neck biogenesis described in more detail below). This matches reports that rhoptry bulb biogenesis occurs first^26,67^, with neck biogenesis not occurring until segmentation. As early as the last mitotic event during early segmentation, rhoptry bulbs were observed as pairs, with 88 of 93 (95%) MTOCs observed forming a mitotic spindle being associated with two RAP1-positive NHS ester densities per MTOC cytoplasmic extension. Finally, in newly invaded ring-stage parasites, strong RAP1 staining was observed at the PPM/PVM (Supplementary Figure 12a), supporting previously reported observations that secreted RAP1 coats the merozoite during invasion^68^.

Our data not only suggests that rhoptry biogenesis occurs well before segmentation, when nuclei still have several rounds of mitosis to complete, but also that rhoptries remain MTOC-associated during these mitotic events. Instances of this association with the MTOC have been observed before^16,67^ but its mechanism remains unknown. To our knowledge, this is the first in-depth documentation of a rhoptry-MTOC association throughout schizogony in *Plasmodium*.

#### Rhoptry heterogeneity during early schizogony and segmentation.

Rhoptries associated with the same MTOC during early schizogony sometimes differ in size (Supplementary Figure 12b). This is not surprising given the speed of mitotic events requires near-constant biogenesis of new rhoptry bulbs. By the time segmentation is underway, instead of inheriting one sister rhoptry in the final mitotic event of schizogony, each MTOC will inherit a pair of rhoptries each. At this point, the speed of these mitotic events slows and parasites reach a point of semi-synchrony. To our surprise, this synchrony does not extend to rhoptry pairs and the two rhoptries inherited by segmenting daughter cells remain different from each other. This heterogeneity in rhoptry pairs during early segmentation has been documented before by electron microscopy^16,67^. Of 109 rhoptry pairs imaged in early segmentation schizonts undergoing their last miotic event (where MTOCs were observed forming mitotic spindles), only 4% had two rhoptries of similar size and density (Supplementary Figure 12d). We observed that 40% of these 109 rhoptry pairs had different size but equal NHS ester density, 21% had the same size but different NHS ester density, and 35% differed in both size and NHS ester density (Supplementary Figure 12e). As expected from previous reports, this heterogeneity was lost after the completion of this last miotic event and rhoptry neck elongation. Of 98 rhoptry pairs imaged in non-mitotic segmenting parasites, 76% had two rhoptries of similar size and density (Supplementary Figure 12d). Overall, these observations of heterogeneity support the hypothesis that rhoptry pairs undergo independent biogenesis rather than splitting off from a single precursor rhoptry. However, it is still unclear how the one-to-two rhoptry transition occurs and whether this heterogeneity has a biological role in rhoptry biogenesis and maturation or whether it is simply an indicator of maturity and a by-product of sequential rhoptry biogenesis.

These observations support a model where rhoptries undergo sequential segregations alongside the MTOC and a second rhoptry forms *de novo* after each segregation event. This process would repeat until the start of segmentation and formation of rhoptry pairs. Constant *de novo* biogenesis could explain why one rhoptry can appear smaller or less mature than the other^16,67^. It is currently unclear, however, why heterogeneity in rhoptry density only appears during early segmentation and not earlier, when biogenesis of new rhoptries is arguably more apparent. Thus, this model is not enough to explain all the variation in rhoptry size and density observed throughout schizogony. Furthermore, a lot of unknowns remain about what exactly governs rhoptry number during the rapid rounds of asynchronous nuclear division^69^, how the transition to a rhoptry pair is signalled, and how many rounds of *de novo* rhoptry formation parasites undergo.

#### Rhoptry neck biogenesis and elongation

In order to observe rhoptry neck biogenesis in more detail, we stained parasites against the rhoptry apical membrane antigen (RAMA, a rhoptry bulb marker) and rhoptry neck protein 4 (RON4, a rhoptry neck marker)^70,71^. RAMA is anchored to the rhoptry bulb membrane and only stains the periphery of the rhoptry bulb as marked by NHS ester (Figure 8b and Supplementary Figure 12c). RON4 is absent from the earliest rhoptry bulbs, appearing as a focus within the rhoptry bulb shortly before early segmentation and before the rhoptry neck could be distinguished from the bulb by NHS ester staining alone (Figure 8b). During early segmentation, when rhoptry pairs first become visible, we observe an uneven distribution of RON4 within each pair. RON4 preferentially associates with one of the rhoptries, with the staining on the second rhoptry being fainter, more diffuse, or even absent in some cases. Of 84 rhoptry pairs observed at this stage, 72 (86%) showed an uneven distribution of RON4. To our surprise, when these rhoptry pairs were of different NHS ester densities, the larger share of RON4 associated with the less dense rhoptry (Figure 8b). Previous observations of rhoptry density differences by electron microscopy have been ascribed to differences in rhoptry age or maturity, with the denser rhoptry being more mature. So, finding RON4 to be more abundant in the less dense rhoptry suggests that either heterogenous RON4 accumulation cannot be explained by rhoptry age or that the less dense rhoptry is instead the older rhoptry. The RON4-positive rhoptry neck elongates during segmentation, attaining its characteristic shape by mid to late segmentation and becoming observable by both RON4 staining and NHS ester (Figure 8). At this point, nearly all rhoptry necks had an equal distribution or RON4. Of 76 rhoptry pairs observed at these stages, 72 (95%) had an equal distribution of RON4.

### The micronemes

**Figure 9:**
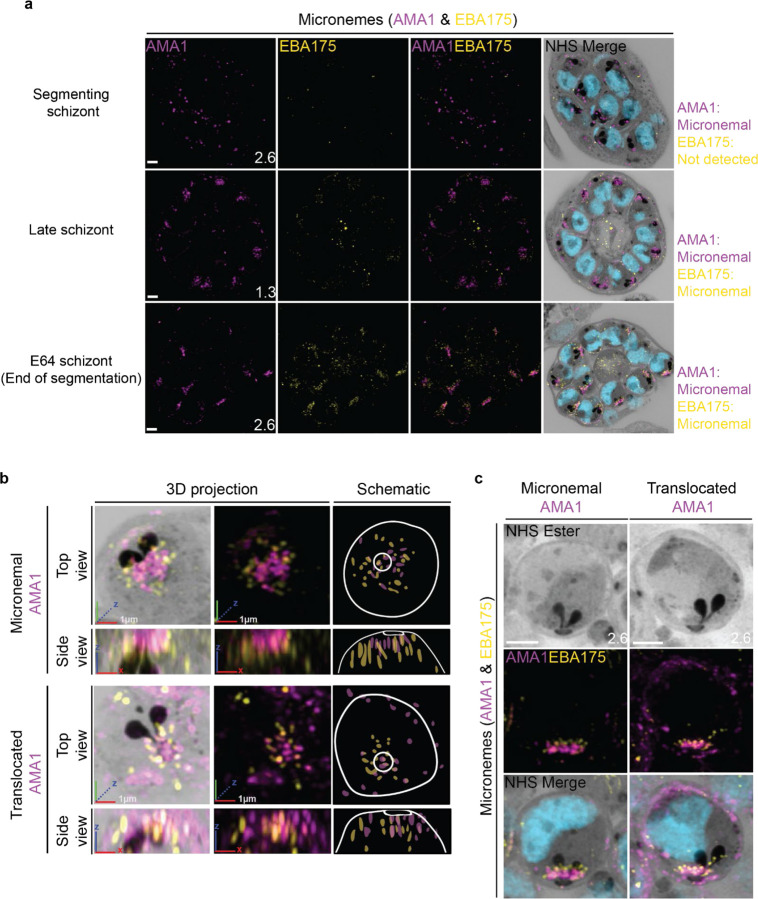
Micronemal proteins AMA1 and EBA175 reside in separate micronemes. **(a)** 3D7 parasites were prepared by U-ExM, stained with NHS ester (greyscale), SYTOX (cyan) and antibodies against the micronemal markers AMA1 (magenta) and EBA-175 (yellow), and imaged using Airyscan microscopy in segmenting schizonts. In schizonts still undergoing segmentation, AMA1 localized to the apical end while EBA175 was not detected. In late schizonts, both EBA175 and AMA1 were present in the micronemes. In E64-arrested schizonts, AMA1 was translocated to the merozoite surface while EBA175 remained micronemal. 3D rendering **(b)** and zooms **(c)** of merozoites with either micronemal or translocated AMA1. Foci of AMA1 and EBA175 to not routinely colocalize with each other. Images are maximum-intensity projections, number on image = Z-axis thickness of projection in µm. White scale bars = 2 µm, RGB scale bars for 3D rendering = 1 µm.

Previous studies have suggested that micronemes may be heterogeneous and that apical membrane antigen 1 (AMA1) and other micronemal markers such as erythrocyte binding antigen-175 (EBA175) reside in different subsets of micronemes^72–74^. We reasoned that individual micronemes may be visible using U-ExM and imaged parasites stained with AMA1 and EBA175 to observe their biogenesis and relative distribution. The first microneme marker to appear during schizogony was AMA1. Large puncta of AMA1 appear near the rhoptries when the basal complex is at its maximum diameter (Figure 9a). At this point, EBA175 is not yet detectable above background fluorescence. At the end of segmentation, we observe AMA1 has arranged itself into small, densely arranged puncta below the APR and around the rhoptry neck. We also observe EBA175 staining in puncta of the same size that are less densely arranged and have little co-localization with AMA1. EBA175 puncta are basal to the AMA1 puncta, being closer to the rhoptry bulb. They also form a cloud of larger diameter than the one formed by AMA1 such that, when viewed from above the APR, two concentric clouds are observed with the core being AMA1 positive and the periphery being EBA175 positive (Figure 9b&c). The AMA1 and EBA175 staining we observed in late-stage schizonts partially overlaps with a punctate NHS-ester pattern, suggesting that NHS-ester stains the micronemes (Figure 9b). However, many NHS-ester-positive foci did not stain with either AMA1 or EBA175 despite being morphologically indistinguishable from those which did. This suggests that NHS-ester stains more than just the micronemes and that some of these foci may be exonemes, dense granules, or other apical vesicles. Alternatively, it is also possible that these additional NHS-ester-positive foci represent micronemes that lack both AMA1 and EBA175.

Using the protease inhibitor E64, we arrested parasites “post-egress” such that AMA1 was translocated. E64 allows for normal daughter cell maturation but prevents RBC plasma membrane rupture once segmentation is complete^75^. We observe that once AMA1 is translocated, the density of apical AMA1 decreases. EBA175, which does not translocate, increases in density, and moves apically, taking the space AMA1 occupied prior to translocation (Figure 9b&c). This is consistent with existing models of sequential microneme translocation and microneme fusion near the APR^76^.

## DISCUSSION

In this work, we apply U-ExM to the asexual blood stage of *Plasmodium falciparum* to provide new insights into the role of the MTOC in establishing apical-basal polarity and in coordinating organelle segregation during schizogony. In the process, we demonstrate U-ExM can be used to study the biogenesis and protein distribution of a variety of organelles and structures within the parasite. Globally, our observations suggest that the MTOC is involved in establishing apical-basal polarity within the parasite and that this polarity is established early in schizogony.

We show that the cytoplasmic extensions of the MTOC appear to act as physical ‘tethers’, connecting the nucleus to parasite plasma membrane (PPM). This creates a space between the PPM and nuclear envelope where we observe multiple parasite structures and organelles including the Golgi, basal complex, rhoptries, and apical polar rings. These structures and organelles remain MTOC-associated despite the constant movement and segregation of MTOCs during mitotic events. Put together, these structures define the apical end of the merozoite. In other systems, MTOC positioning has been described as a source of cellular polarity, polarizing a cell by directing cargo of secretory organelles to a defined area ^77–79^. While this has not been previously described in *Plasmodium*, this model fits well with our observation of the Golgi being adjacent to the MTOC throughout schizogony. This Golgi positioning could also perhaps provide a region of local cytoplasmic exclusion that promotes biogenesis of rhoptries, micronemes, dense granules, and IMC, which are all at least partially formed by Golgi-derived cargo^26,76,80,81^. While the biogenesis of the apical polar rings seems to also be coupled to the MTOC, we do not yet know how they are nucleated or whether they are also dependent on Golgi-derived cargo.

This study also shows MTOC-coupled organelle segregation and biogenesis to an extent never observed before. Specifically, we observe that the Golgi, rhoptries, and basal complex match the MTOC cytoplasmic extensions in number throughout early schizogony and segregate with MTOCs during the mitotic events of schizogony. As this study used fixed cells, we lack the temporal power to precisely define the order of these events. Using known markers of parasite age, however, we describe putative transition states that nascent rhoptries and basal complexes adopt when being segregated with MTOCs. We put forward a model where cytoplasmic MTOC-associated events occur before intra-nuclear MTOC-associated events. That is, the duplication of the MTOC cytoplasmic extension and organelles precedes MTOC duplication and the formation of a mitotic spindle such that this cytoplasmic duplication represents the first identifiable step in the commitment of a nucleus to the next round of mitosis. Interestingly, nuclei can sometimes have MTOCs forming a mitotic spindle that have already committed to the next round of mitosis by duplicating their cytoplasmic cargo. That is, a mitotic nucleus can show four cytoplasmic extensions and sets of apical organelles at the same time. While this supports our hypothesis that duplication of apical organelles and cytoplasmic MTOC components happens upstream from intra-nuclear mitotic events, it does not tell us how this relates to the genome copy number inside the nucleus. So, we cannot say whether DNA replication and associated checkpoints occur upstream or downstream from the cytoplasmic events that seem to commit an MTOC to mitosis.

Lastly, we contribute important evidence toward hypotheses across several open questions regarding organelle biogenesis and segregation in *Plasmodium*. We observe contacts between the MTOC and both the mitochondria and apicoplast during fission that suggest a role for this structure in monitoring copy number of these organelles. We also document sequential fission events in both the apicoplast and mitochondria that are suggestive of branching point fission. Lastly, we see temporal and spatial localization patterns of AMA1, EBA175, and RON4 that support theories of heterogeneity within the apical organelles.

U-ExM represents an affordable and adaptable sample preparation method that can be applied to any microscope to produce images with far greater visible detail than conventional light microscopy platforms. Applying this technique to the visualization of organelle biogenesis and segregation throughout schizogony allowed us to observe these processes in the context of structures that could previously only be investigated in detail using electron microscopy. The flexibility and scalability of this technique allowed us to image more than 600 individual parasites at a variety of developmental stages, increasing the confidence of our observations and giving them some temporal resolution. To our knowledge, this paper represents the most comprehensive study of a single organism using U-ExM, with a total of 13 different subcellular structures investigated.

Our inability to pinpoint the nucleation site of the SPMTs or resolve the plasma membrane from the IMC highlights some of the limitations of U-ExM as applied in this work. The subpellicular network that holds IMC1g and lines the cytoplasmic face of the IMC sits approximately 20 nm below the parasite surface, where we would find MSP1^82^. Thus, the distance between IMC1g and MSP1 post-expansion is around 90nm. This is below our imaging resolution with Airyscan2, and close to the maximum resolution we could achieve through other super-resolution methods compatible with our current setup when antibody effects are considered. Thus, some parasite structures remain beyond the resolution achieved in this study. In order to resolve the IMC from the plasma membrane or resolve multiple apical polar rings using light microscopy, we would need to employ single molecule localization microscopy or iterative expansion microscopy^83^, which increases expansion factor to ~20x.

We also noticed some drawbacks and artifacts introduced by U-ExM. Most visually striking was that the hemozoin crystal of the food vacuole does not expand^84^, which leaves a large space that lacks NHS ester staining in the center of the parasite. For nearly all antibodies used in this study, significant off-target fluorescence was observed inside the food vacuole. Thus, U-ExM may not be as useful for studying food vacuole biology. Occasionally, significant SYTOX (DNA stain) fluorescence was observed at either the nuclear envelope or parasite plasma membrane. It is unclear if this represents an expansion-induced artifact or a PFA-fixation artifact that is only now observable. Lastly, for cells stained with Mitotracker, some non-specific background was observed that seemed to correlate with protein density as observed by NHS-ester.

Malaria parasites have been extensively studied using electron microscopy to determine their ultrastructure and live-cell microscopy to observe their most dynamic processes in real time. Much of what we uncovered in this study involved dynamic processes that are too small to be resolved using conventional live-cell microscopy. Specifically, we made important observations about *P. falciparum* organelle biogenesis and the organization of *Plasmodium* cell division around the MTOC. Rather than a replacement for any existing microscopy techniques, we see U-ExM as a complement to the suite of techniques available to study the cell biology of malaria parasites, which bridges some of the limitations of electron microscopy and live-cell microscopy. As such, there are many parasite processes that are logical candidates for investigation by U-ExM. Some of these have been highlighted in this paper, but others remain completely unexplored. Merozoite invasion, for example, has been well-studied using a variety of microscopy techniques^31,68,85–87^, but U-ExM would allow us to visualise how all the apical organelles associate with each other and rearrange in three-dimensions across the established time-course^86^ of this process. Another logical candidate for investigation by U-ExM is the rapid disassembly of the IMC and other merozoite organelles immediately following invasion^88^, a process where the parasite undergoes rapid and dramatic morphological rearrangements that lie beyond the resolution of live-cell microscopy.

## MATERIALS AND METHODS

### *Plasmodium falciparum* culture

Unless otherwise stated, all parasites in this study were 3D7-Cas9^41^. For imaging of the apicoplast, the previously generated ACP-transit-peptide-GFP cell line was used ^56^. For imaging of Kelch13, the previously generated 2xFKBP-GFP-K13 parasites were used^63^.

All parasites were cultured in O^+^ human red blood cells at 4% haematocrit in RPMI-1640 containing 25 mM HEPES, 50 mg/L hypoxanthine and 0.5% w/v Albumax II ^89^. All parasite cultures were incubated on a shaker at 37 °C in a gas mixture of 1% O_2_, 5% CO_2_, and 94% N_2_ as previously described. The smHA-tagged Pf3D7_0311800 (ATP Synthase F0 Subunit D) cell line was maintained under selection of 5 nm WR99210. Apicoplast targeting signal-GFP line was maintained under selection of x mm x. 2xFKBP-GFP-K13 parasites were maintained under selection of 0.9 µm DSM1.

Parasites were routinely synchronised using sorbitol lysis. Briefly, parasite cultures were resuspended in 5% w/v D-sorbitol, resulting in the selective lysis of schizont-stage parasites^90^.

For samples where parasites were arrested as schizonts using either trans-Epoxysuccinyl-L-leucylamido(4guanidino)butane (E64)^91^ or compound 1 (C1)^92^, late schizont-stage cultures were treated with either 10 µm E64 for ~3h or 5 µm C1 for ~5 hours.

### Plasmid generation and transfection

For imaging of the mitochondria, a cell line where ATP-Synthase F0 Subunit D (Pf3D7_0311800) had a C-terminal spaghetti-monster HA tag^35^ was generated (Supplementary Figure 10). To create the Pf3D7_0311800 smHA HDR plasmid, the 3D7_0311800 5’ and 3’ homology regions were PCR amplified from 3D7 genomic DNA with oligonucleotides oJDD4893/oJDD4894 and oJDD4891/oJDD4892, respectively. The two pieces were fused together using Sequence Overlap Extension PCR (SOE PCR) using oJDD4891/oJDD4894 and the piece was digested with NotI/XhoI and ligated with T4 ligase to generate pSAB55. To create the PF3D7_0311800 targeting guide RNA plasmid, oJDD4889/oJDD4890 were annealed, phosphorylated, and ligated into BpiI-digested pRR216 to generate pSAB81. All oligonucleotide sequences are shown in Supplementary Table 1.

For transfection, 100 µg of pSAB55 plasmid was linearized with StuI and transfected into 3D7-Cas9, along with 100 µg of pSAB81. A day following transfection, parasites were treated with 5 nm WR99210 until 13 days, when resistant parasites were detected.

### Ultrastructure expansion microscopy

Ultrastructure expansion microscopy (U-ExM) was performed as previously described with minor modification^3,5,6^. 12 mm round Coverslips (Fisher Cat. No. NC1129240) were treated with poly-d-lysine for 1 h at 37 °C, washed twice with MilliQ water, and placed in the wells of a 12-well plate. Parasite cultures were set to 0.5% haematocrit, and 1 mL of parasite culture was added to the well containing the coverslip and for 15 min at 37 °C. Culture supernatants were removed, and cultures were fixed with 1 mL of 4% *v*/*v* paraformaldehyde (PFA) in 1xPBS for 15 min at 37 °C. For some experiments visualising cytostomes, cultures were instead fixed in 4% v/v PFA + 0.01% v/v glutaraldehyde in 1xPBS. Following fixation, coverslips were washed three times with 37 °C PBS before being treated with 1 mL of 1.4 % *v*/*v* formaldehyde/2% *v*/*v* acrylamide (FA/AA) in PBS. Samples were then incubated at 37 °C overnight.

Monomer solution (19% *w*/*w* sodium acrylate (Sigma Cat. No. 408220), 10% *v*/*v* acrylamide (Sigma Cat. No. A4058, St. Louis, MO, USA), 2% *v*/*v* N,N’-methylenebisacrylamide (Sigma Cat. No. M1533) in PBS) was typically made the night before gelation and stored at −20 °C overnight. Prior to gelation, FA/AA solution was removed from coverslips, and they were washed once in PBS. For gelation, 5 µL of 10% v/v tetraethylenediamine (TEMED; ThermoFisher Cat. No. 17919) and 5 µL of 10% *w*/*v* ammonium persulfate (APS; ThermoFisher Cat. No. 17874) were added to 90 µL of monomer solution and briefly vortexed. Subsequently, 35 µL was pipetted onto parafilm and coverslips were placed (cell side down) on top. Gels were incubated at 37 °C for 30 minutes before being transferred to wells of a 6-well plate containing denaturation buffer (200 mM sodium dodecyl sulphate (SDS), 200 mM NaCl, 50 mM Tris, pH 9). Gels were incubated in denaturation buffer with shaking for 15 minutes, before separated gels were transferred to 1.5 mL tubes containing denaturation buffer. 1.5 mL tubes were incubated at 95 °C for 90 minutes. Following denaturation, gels were transferred to 10 cm Petri dishes to containing 25 mL of MilliQ water for the first round of expansion and placed onto a shaker for 30 minutes three times, changing water in between. Gels were subsequently shrunk with two 15-minute washes in 25 mL of 1x PBS, before being transferred to 6-well plates for 30 minutes of blocking in 3% BSA-PBS at room temperature. After blocking, gels were incubated with primary antibodies, diluted in 3%BSA-PBS, overnight. After primary antibody incubation, gels were washed three times in 0.5% v/v PBS-Tween 20 for 10 minutes before incubation with secondary antibodies diluted in 1x PBS for 2.5 hours. Following secondary antibody incubation, gels were again washed three times in PBS-Tween 20, before being transferred back to 10 cm Petri dishes for re-expansion with three 30-minute MilliQ water incubations.

Gels were either imaged immediately following re-expansion, or stored in 0.2% w/v propyl gallate in MilliQ water until imaging. For gels stained with BODIPY TR Ceramide, the fully expanded gel was incubated overnight at room temperature in 0.2% w/v propyl gallate (Acos Organics Cat No. 131581000) solution containing BODIPY TR ceramide (2 µM final concentration).

For parasites stained with Mitotracker Orange CMTMRos (ThermoFisher, M7510), parasite cultures were resuspended in incomplete media + bicarb containing 300 nM Mitotracker Orange CMTMRos. Parasites cultures were then stained with Mitotracker for 35 minutes while settling on Poly-d-lysine coated coverslips. From this point, the expansion protocol was followed as described above, with the exception that all steps when possible were carried out protecting the sample from light.

#### Cryopreservation and thawing of gels.

A proportion of gels imaged in this study were cryopreserved and subsequently thawed prior to imaging^83^. Gels were frozen either unstained, following the first round of expansion, or frozen stained, following the second round of expansion. To freeze, a portion of the expanded gel was placed into a 6-well dish and washed three times with 50% glycerol in MilliQ water for 30 minutes. Fresh glycerol was then added, and the gels were stored at −20 °C for future use. To thaw unstained gels, the glycerol was replaced with MilliQ water and incubated at room temperature for 30 minutes. Gels were then washed and shrunk with three 20-minute washes in 1x PBS at room temperature before proceeding with the antibody staining process normally. Stained gels were thawed with three washes in MilliQ water for 30 minutes before proceeding with imaging as normal.

### Stains and antibodies

A comprehensive list of all stains and antibodies used in this study, their working concentrations, and source(s) can be found in Supplementary Table 2.

### Image acquisition

All images presented in this study were taken using either a Zeiss LSM800 AxioObserver with an Airyscan detector, or a Zeiss LSM900 AxioObserver with an Airyscan 2 detector. Imaging on both microscopes was conducted using a 63x Plan-Apochromat objective lens with a numerical aperture of 1.4. All images were acquired as Z-stacks that had an XY pixel size of 0.035 µm and a Z-slice size of 0.13 µm.

### Image analysis

#### Image processing and presentation

All images were Airyscan processed using 3D processing at moderate filter strength on ZEN Blue Version 3.1 (Zeiss, Oberkochen, Germany).

The majority of images presented in this study are presented as z-projections for viewing purposes. The majority of images are presented as maximum intensity projections, but those that contain BODIPY TR Ceramide are presented as average intensity projections for viewing and interpretation purposes.

#### 3D rendering

3D renderings of micronemal proteins AMA1 and EBA175 were produced using the 3D analysis package on ZEN Blue version 3.5.

#### Measurement of interpolar spindles and subpellicular microtubules

All length measurements reported in this study were obtained using the “Measure 3D distance” function of ZEN Blue Version 3.1. The length of Interpolar spindle microtubules and subpellicular microtubules was determined as the 3D distance between the start and end points of continuously stained stretches of anti-tubulin staining. Interpolar microtubules were defined as those whose staining appeared to contact both MTOCs as defined by NHS ester, while non-interpolar microtubules were those that did not meet these criteria. Subpellicular microtubules were only measured in C1-arrested schizonts that had visibly completed segmentation based on the basal complex as visualised by NHS ester. Any microtubule that did not appear connected to the apical polar ring, or extend towards the basal complex, was excluded from the analysis. Cell diameter was defined as the greatest XY distance on any z-slice between two points of the parasite as defined by NHS Ester staining. Merozoite length was defined as the 3D distance between the center of the apical polar rings and basal complex as defined by NHS ester staining.

#### Apicoplast and mitochondria area analysis

All area measurements presented were obtained using the “Area” function on ZEN Blue Version 3.1. Images were presented as a maximum intensity projection before free hand outlining the apicoplast or the mitochondria in each image. The sum of all fragments was then calculated to find the total area of the organelles per cell.

#### Mitochondria residual body analysis

In C1-arrested schizonts, the proportion of total mitochondria staining found in the residual body was calculated as follows. Using ZEN Blue version 3.1, a maximum intensity projection of the entire cell was generated and based on the NHS staining, the entire parasite was defined as the region of interest. Signal intensity of the channel staining the mitochondria was calculated inside the full cell, which was defined as total mitochondria fluorescence. Subsequently, the residual body was defined the area within the parasite vacuole, but external to all merozoite plasma membranes, as based on BODIPY TR ceramide staining. A second maximum intensity projection of this subsection of the schizont was made, the residual body was defined as the region of interest and this signal intensity inside this region of interest was defined as residual body mitochondria fluorescence.

To determine residual body mitochondria fluorescence (RB) as % total mitochondria fluorescence (total), the following equation was used:

$$\left(\frac{RB\;fluorescence}{Total\;fluorescence}\right)\times 100$$

In each of the parasites included in this analysis, the number of merozoites was defined as the number of distinct nuclei as determined by SYTOX staining. To determine residual body mitochondria fluorescence as % of one merozoite, the following equation was used:

$$\left(RB\;fluorescence÷\left(\frac{Total\;fluorescence-RB\;fluorescence}{Number\;of\;merozoites}\right)\right)\times 100$$

Cells where no mitochondria fluorescence was visible inside the residual body, while visible inside merozoites, were defined as having no residual body mitochondria. An attempt was made to do a similar analysis on apicoplast stained cells, but no visible apicoplast staining was ever observed in the residual body.

### Statistical analysis

#### Estimation of actual distance from expanded samples

Expansion factors for 43 gels used in this study were determined as follows. Gels were assumed to have an initial diameter of 12 mm, as they are formed on a 12 mm diameter coverslip. Gels were subsequently measured following expansion to the nearest whole millimetre, and the expansion factor was defined as the expanded gel diameter divided by the initial gel diameter (12 mm). Gels whose edges were damaged or malformed, and therefore their diameters could not be actually measured, were excluded. Gels in this study had a median expanded diameter of 51 mm, which corresponds to a median expansion factor of 4.25 (Supplementary Figure 7)

#### Generation of graphs and statistical analysis

All graphs presented in this study were generated using GraphPad PRISM 9. All error bars in this study represent standard deviation. Differences between samples analysed by ANOVA was determined as difference where the p-value was <0.05. For scatterplots, slopes were considered significantly non-zero when the p-value was <0.05.

## Supplementary Material

Supplement 1

## Figures and Tables

**Figure 10:**
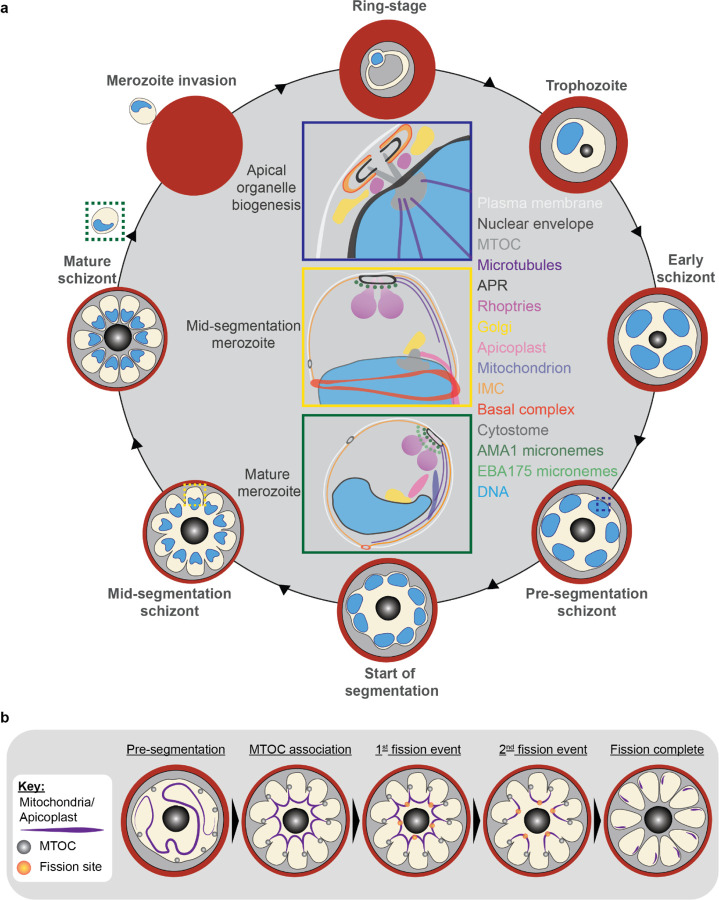
Summary of organelle organization and fission during schizogony. **(a)** Apical organelle biogenesis: Biogenesis of the rhoptries, Golgi, basal complex, and apical polar rings occur at the cytoplasmic extensions of the nuclear MTOC, between the nuclear envelope and parasite plasma membrane. Duplication and segregation of these organelles appears to be tied to MTOC duplication and segregation following nuclear divison. Mid-segmentation merozoite: The rhoptry neck is distinguishable from the bulb, and AMA1-positive micronemes are present at the apical end of the forming merozoite. Each merozoite has inherited a cytostome. Subpellicular microtubules stretch the entire distance from the apical polar rings and the basal complex. The apicoplast has attached to the nuclear MTOC and begun fission. Mature merozoite: The parasite has completed segmentation, and each merozoite contains a full suite of organelles. The nuclear MTOC is no longer visible, and EBA175-positive micronemes are both visible and separate from AMA1-positive micronemes. **(b)** Model for fission of the mitochondrion and apicoplast. Prior to fission, both the apicoplast and mitochondrion branch throughout the parasite cytoplasm, before associating with the MTOC of each forming merozoite. For the apicoplast, this occurs during the final mitosis, but not until late in segmentation for the mitochondrion. Following MTOC association, the apicoplast and mitochondrion undergo a first fission event, which leaves an apicoplast and mitochondrion shared between forming merozoite pairs. Subsequently both organelles undergo a second fission event, leaving each forming merozoite with a single apicoplast and mitochondrion.
