# Supplementary material for: Atlas of Plasmodium falciparum intraerythrocytic development using expansion microscopy
Source: bioRxiv. 2023 Oct 9:2023.03.22.533773. Originally published 2023 Mar 24. Preprint. [Version 3] doi: 10.1101/2023.03.22.533773 (PMC10055389; doi:10.1101/2023.03.22.533773)
Supplement: Supplement 1 — Figure 1 – Figure Supplement 1: Size of gels imaged in this study. (a) 42 of the expanded gels in this study were measured post-expansion to (b) calculate their expansion factor. The median gel length was 51 mm, which corresponds to a 4.25x expansion factor, and so for ‘actual’ measurements, values were divded by 4.25. Figure 1 – Figure Supplement 2: Cytoplasm staining during intraerythrocytic development. 3D7 parasites were prepared by U-ExM, stained with NHS Ester (greyscale), BODIPY TRc (white), SYTOX (cyan) and anti-aldolase (cytoplasm; magenta) antibodies and imaged by Airyscan microscopy across the asexual blood-stage. Yellow line indicates likely position of food vacuole lacking hemozoin crystal. Images are maximum-intensity projections, number on image = Z-depth in μm of projection. Scale bars = 2 μm. Figure 2 – Figure Supplement 1: Characterisation of outer centriolar plaque branches. (a) Mononucleated 3D7 parasites were prepared by U-ExM, stained with NHS Ester (greyscale), anti-centrin (outer centriolar plaque (CP), magenta) and anti-tubulin (microtubules, yellow) antibodies and imaged by Airyscan microscopy. Nuclei form the first hemispindle before centrin is observed, and associate with the parasite plasma membrane (PPM) following visualisation of centrin. (b) Quantification of the number of cytoplasmic extensions (branches) per outer CP in parasites of varying age. Indvidual centriolar plaques from mitotic spindles were assessed separately. (c) Quantification of the co-occurrence of outer CP branch number with centrin foci. (d) In the 8% of 2 cytoplasmic extension outer CPs that had only a single centrin focus, these were likely non-resolvable at the image resolution. (e) Parasites that had started mitosis were stained with NHS Ester and Golgi marker ERD2 (magenta). Golgi was seen associating with centriolar plaques and appeared to duplicate with the formation of the mitotic spindle. Images are maximum-intensity projections, number on image = Z-depth i [file media-1.pdf]

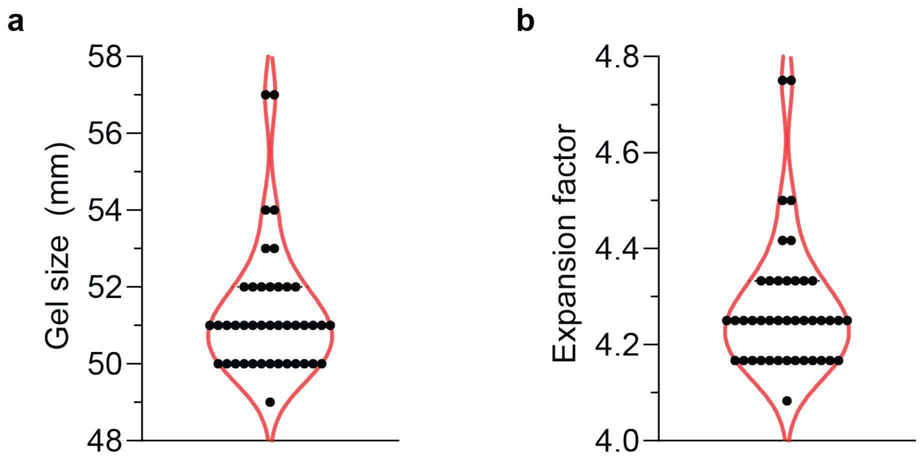

Figure 1 – Figure Supplement 1

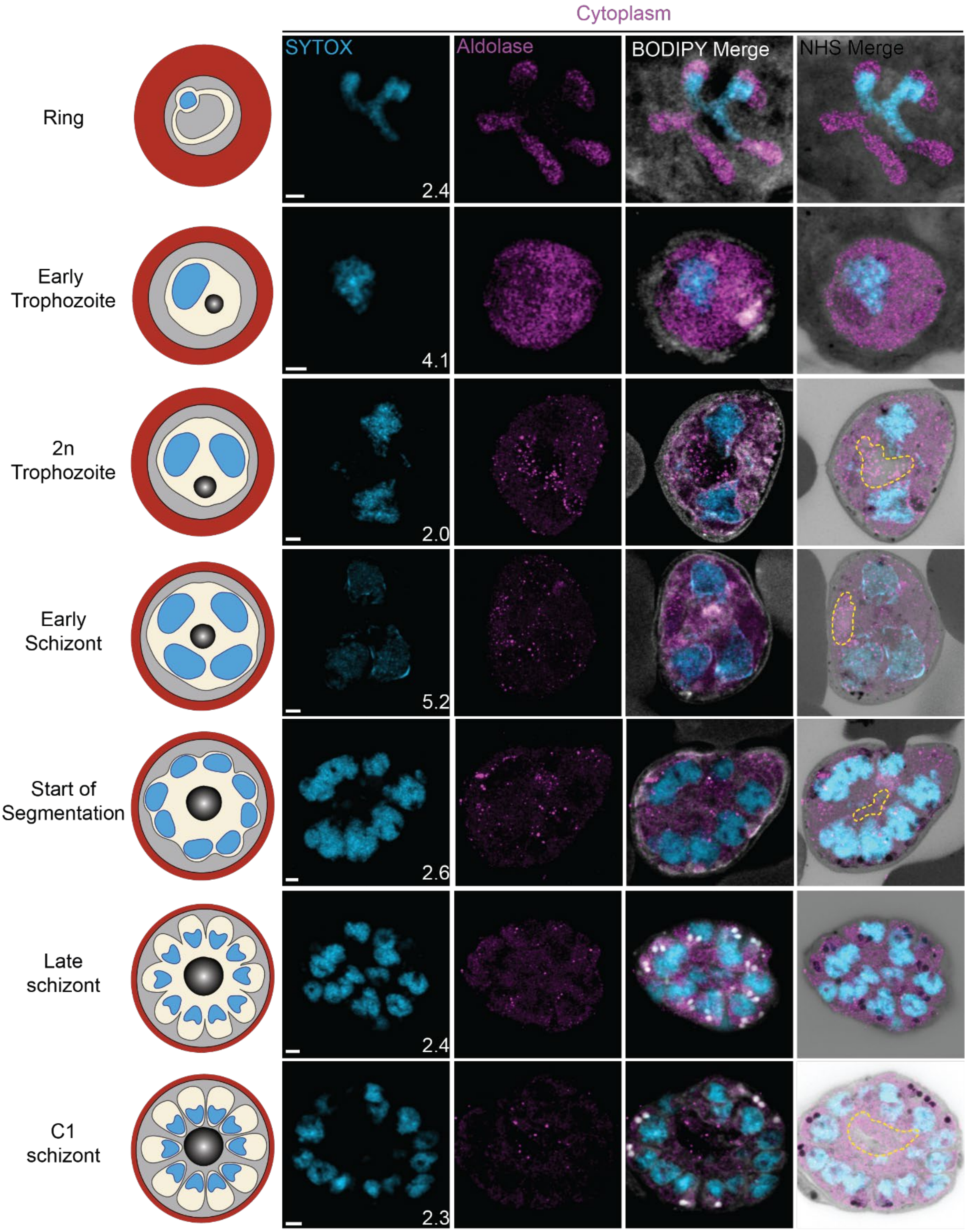

Figure 1 – Figure Supplement 2

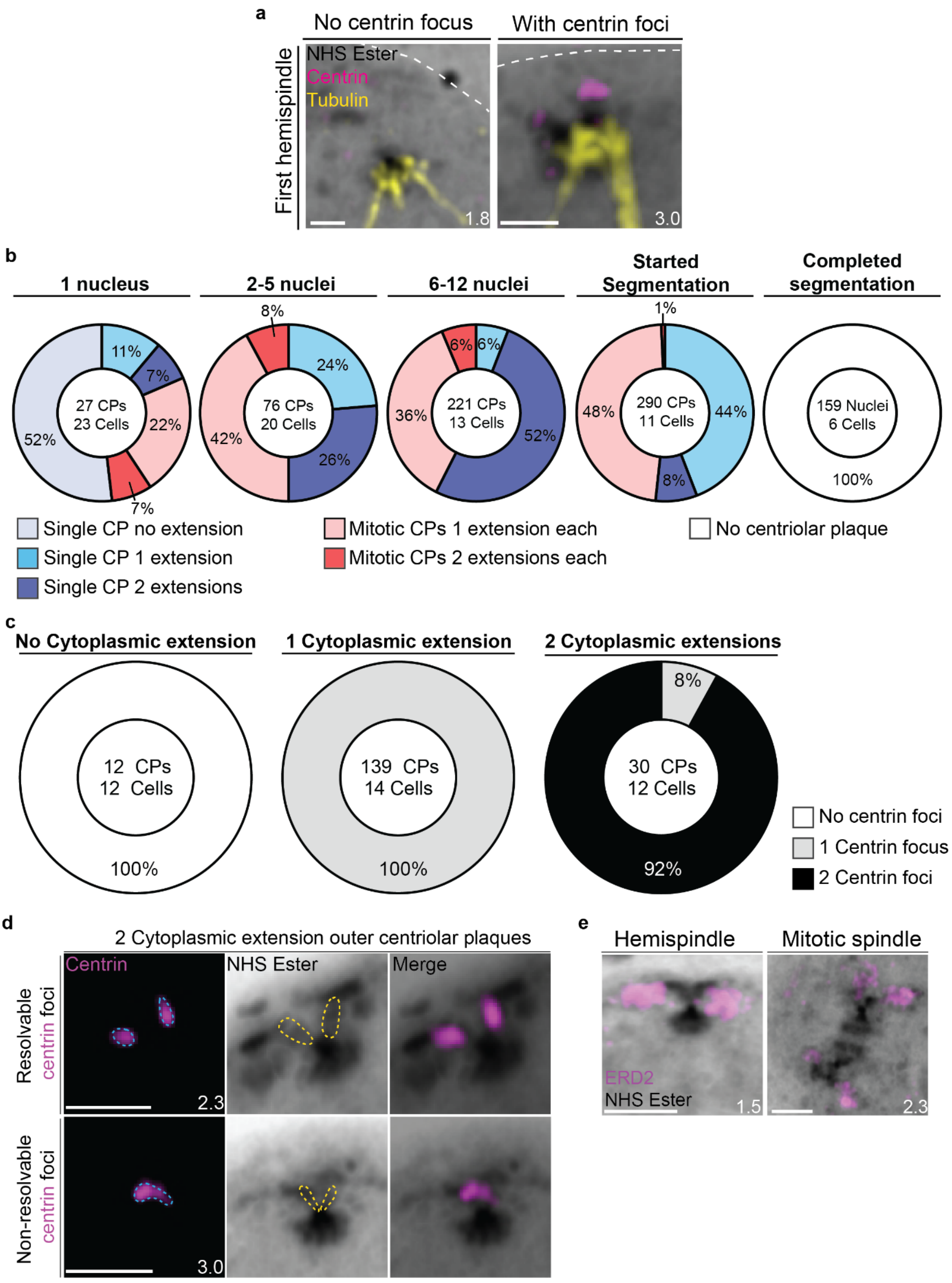

Figure 2 – Figure Supplement 1

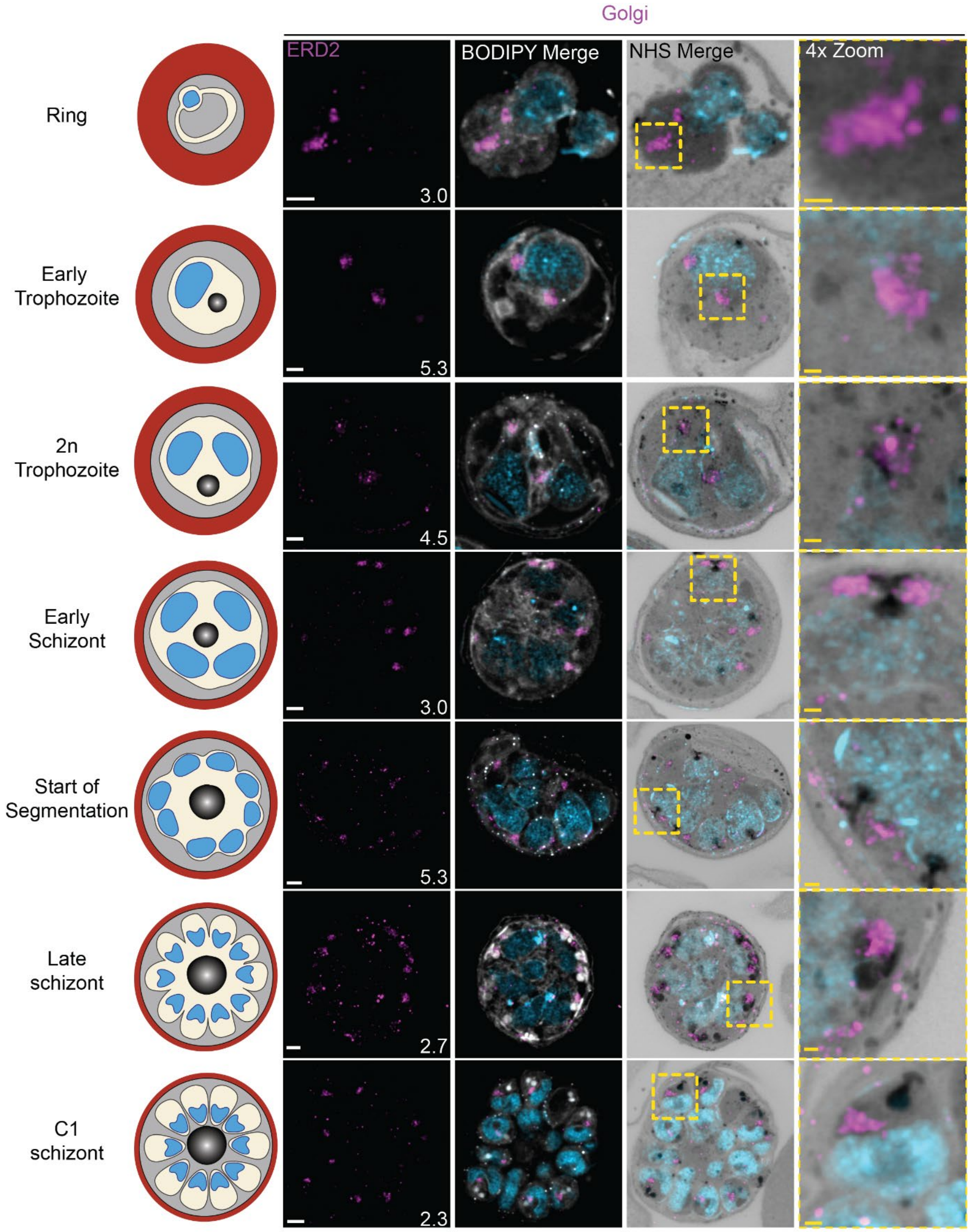

Figure 2 – Figure Supplement 2

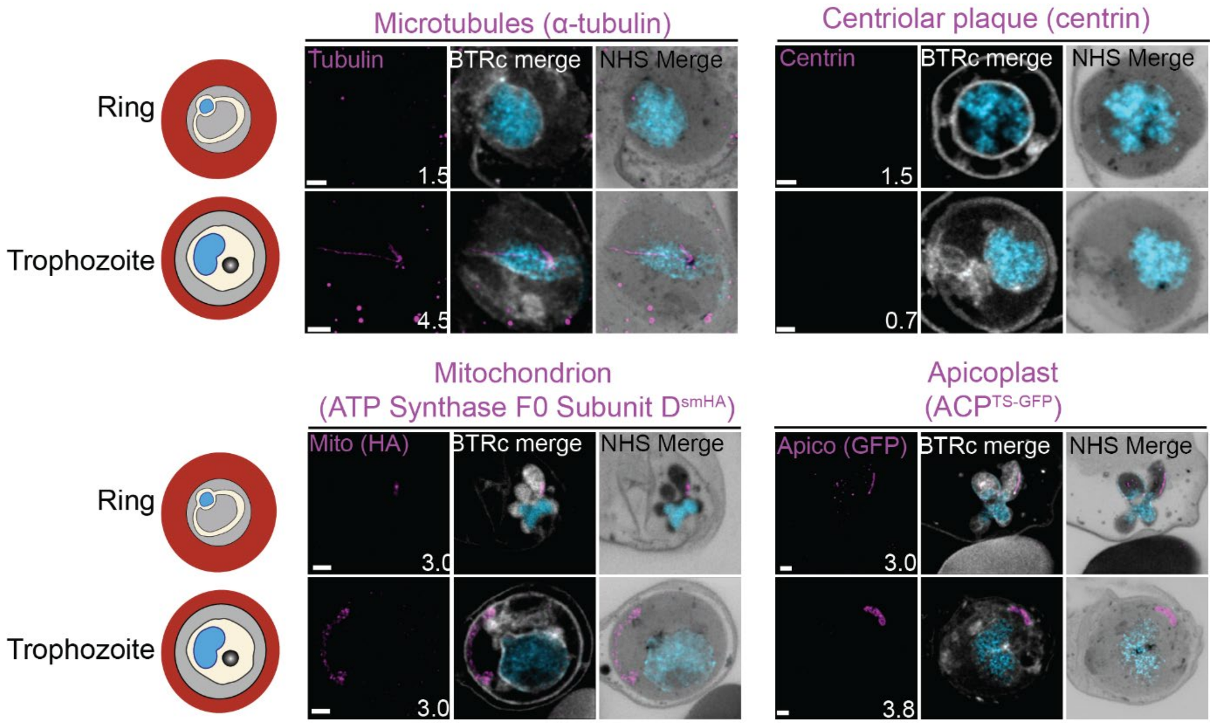

Figure 3 – Figure Supplement 1

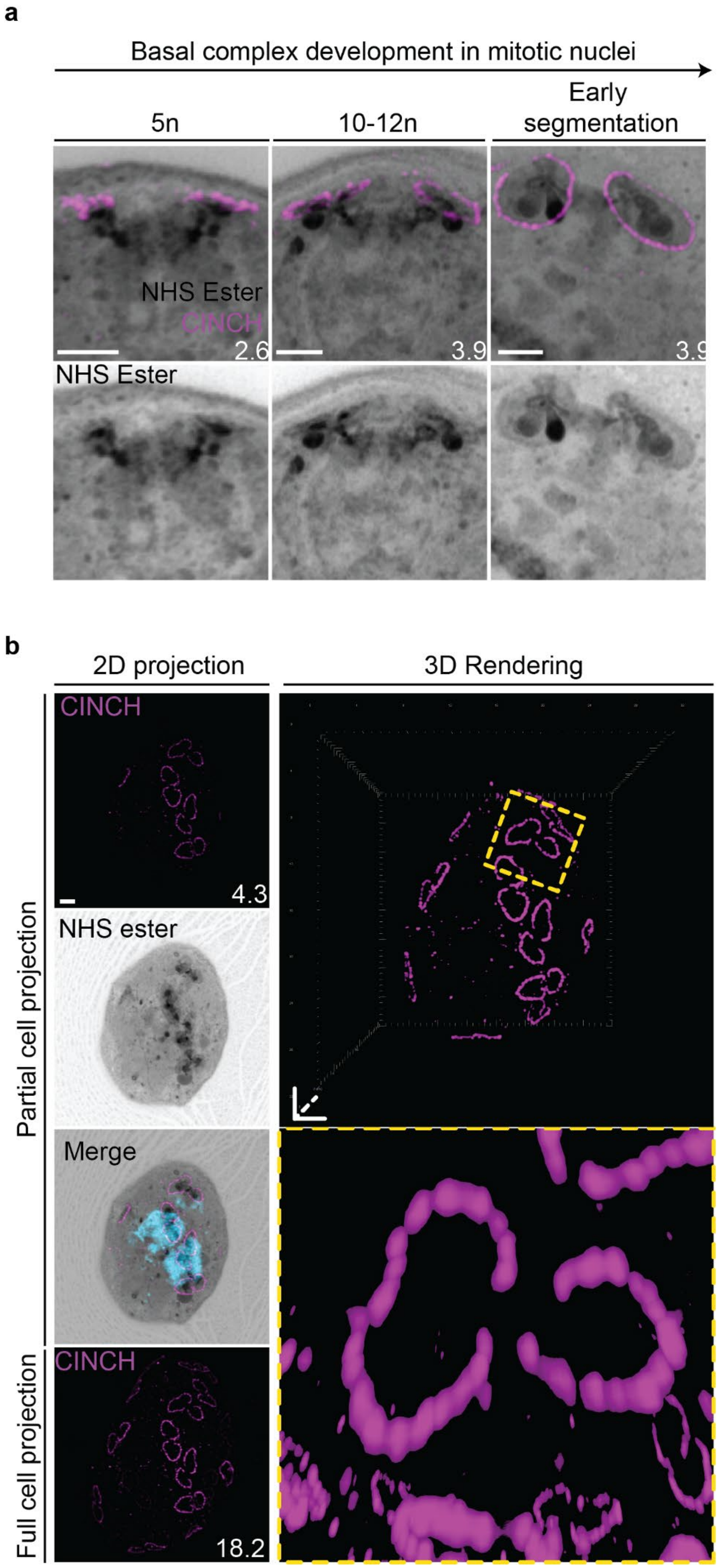

Figure 4 – Figure Supplement 1

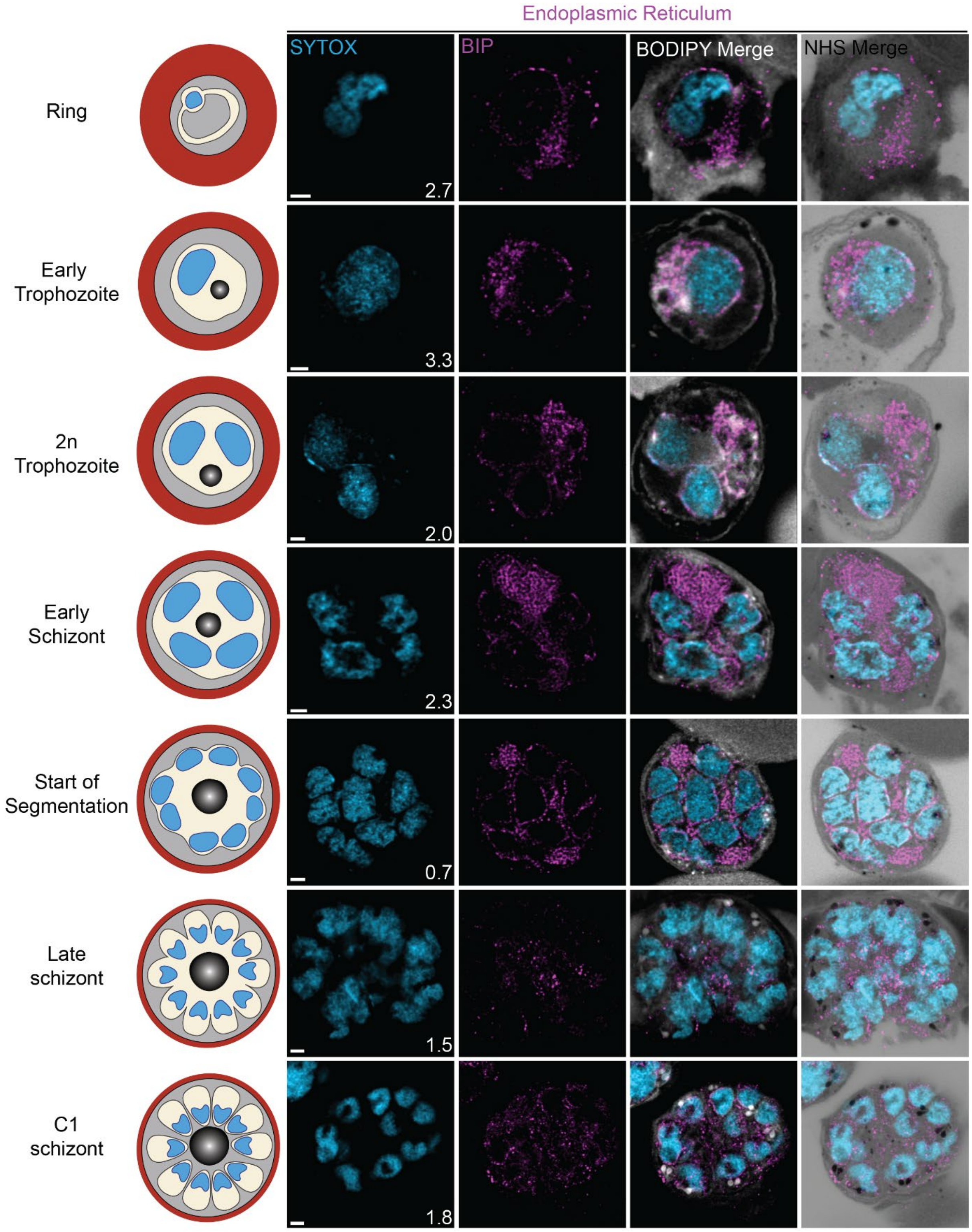

Figure 4 – Figure Supplement 2

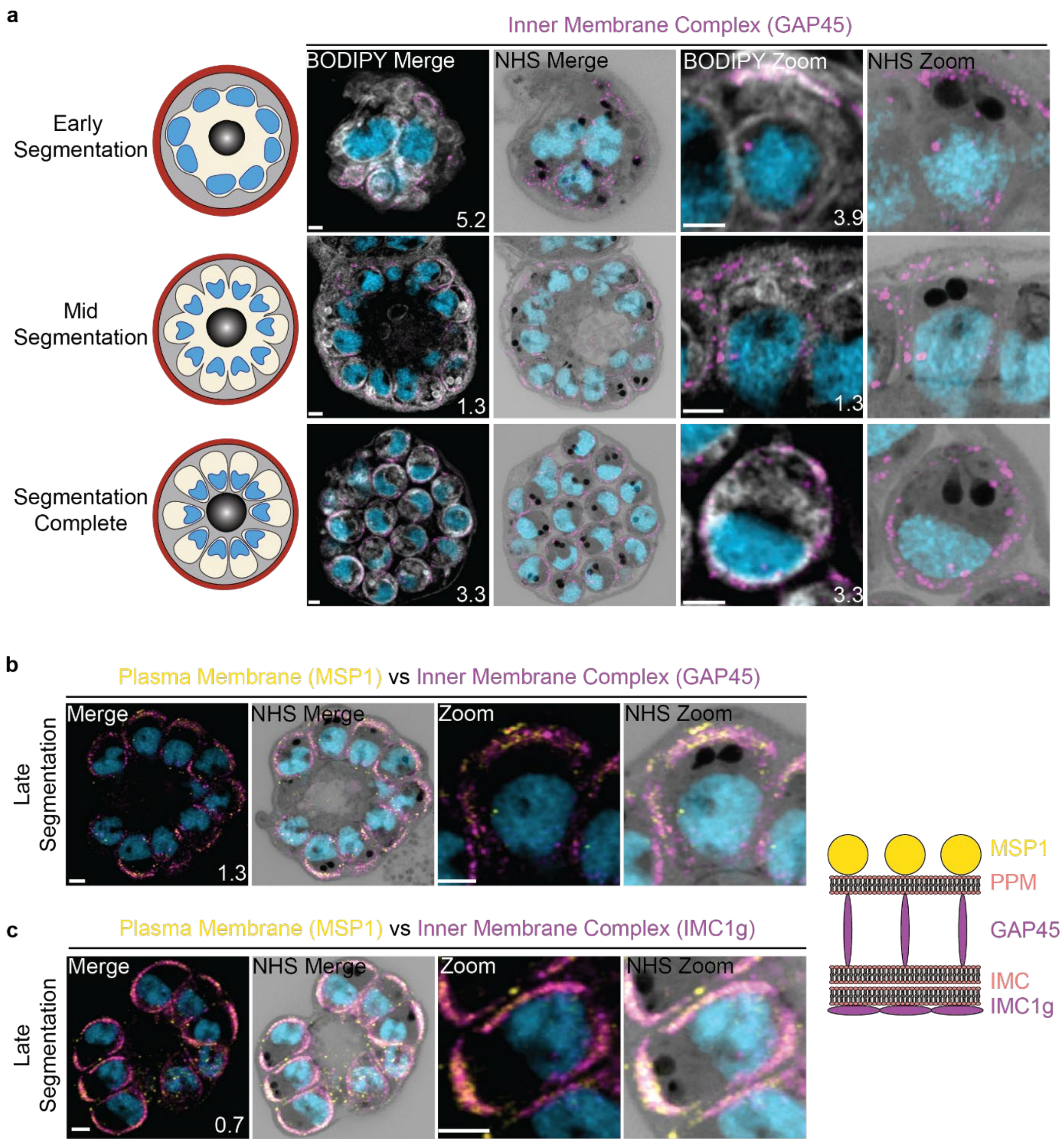

Figure 4 – Figure Supplement 3

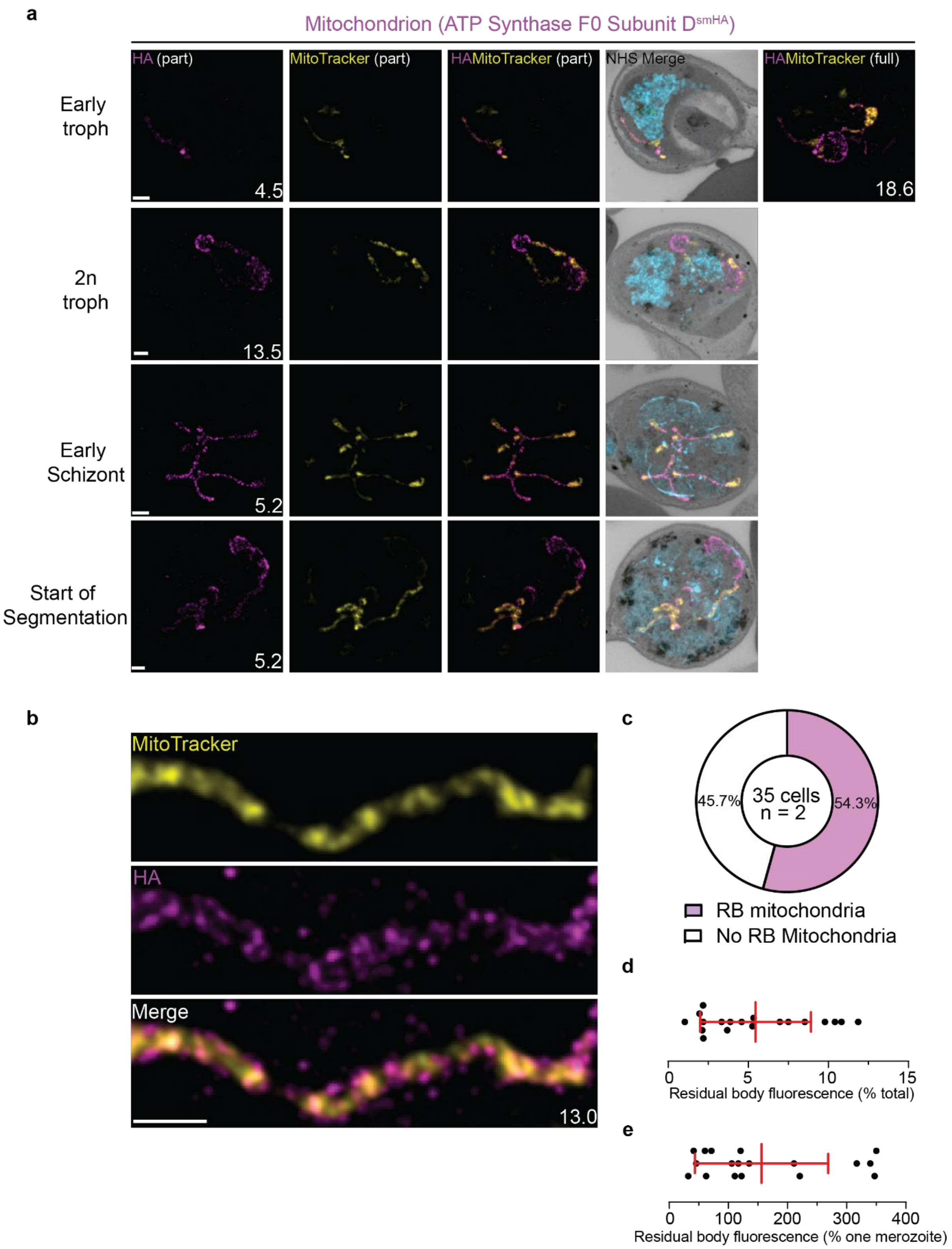

Figure 5 – Figure Supplement 1

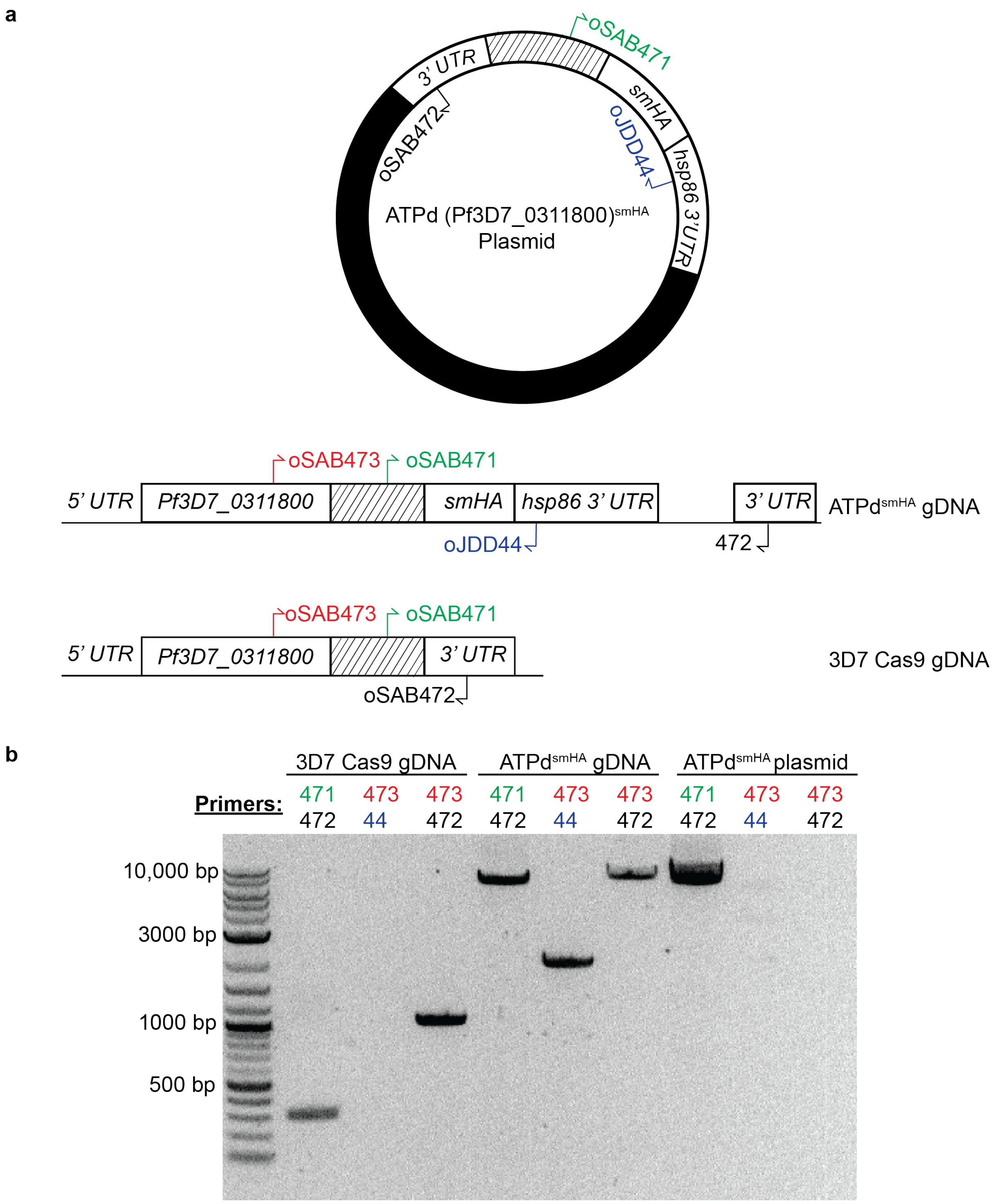

	471/472	473/44	473/472
3D7 Cas9 gDNA	321 bp	--------	1038 bp
ATPd^{smHA} gDNA	6890 bp	2017 bp	8008 bp
ATPd^{smHA} plasmid	6890 bp	--------	--------

Figure 5 – Figure Supplement 2

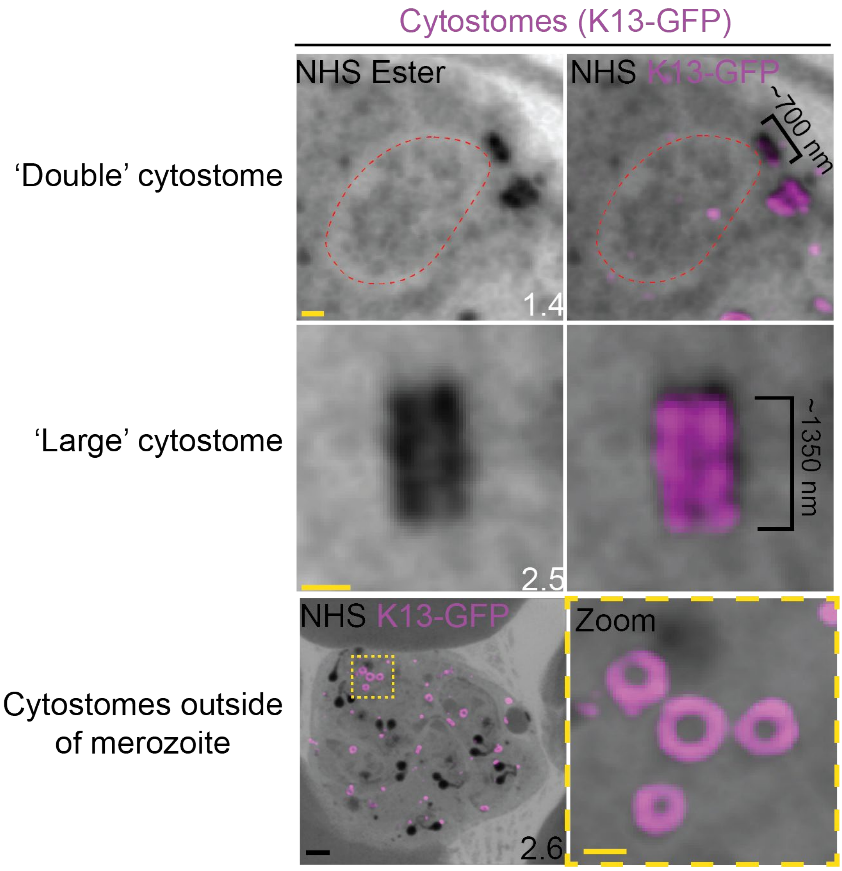

Figure 7 – Figure Supplement 1

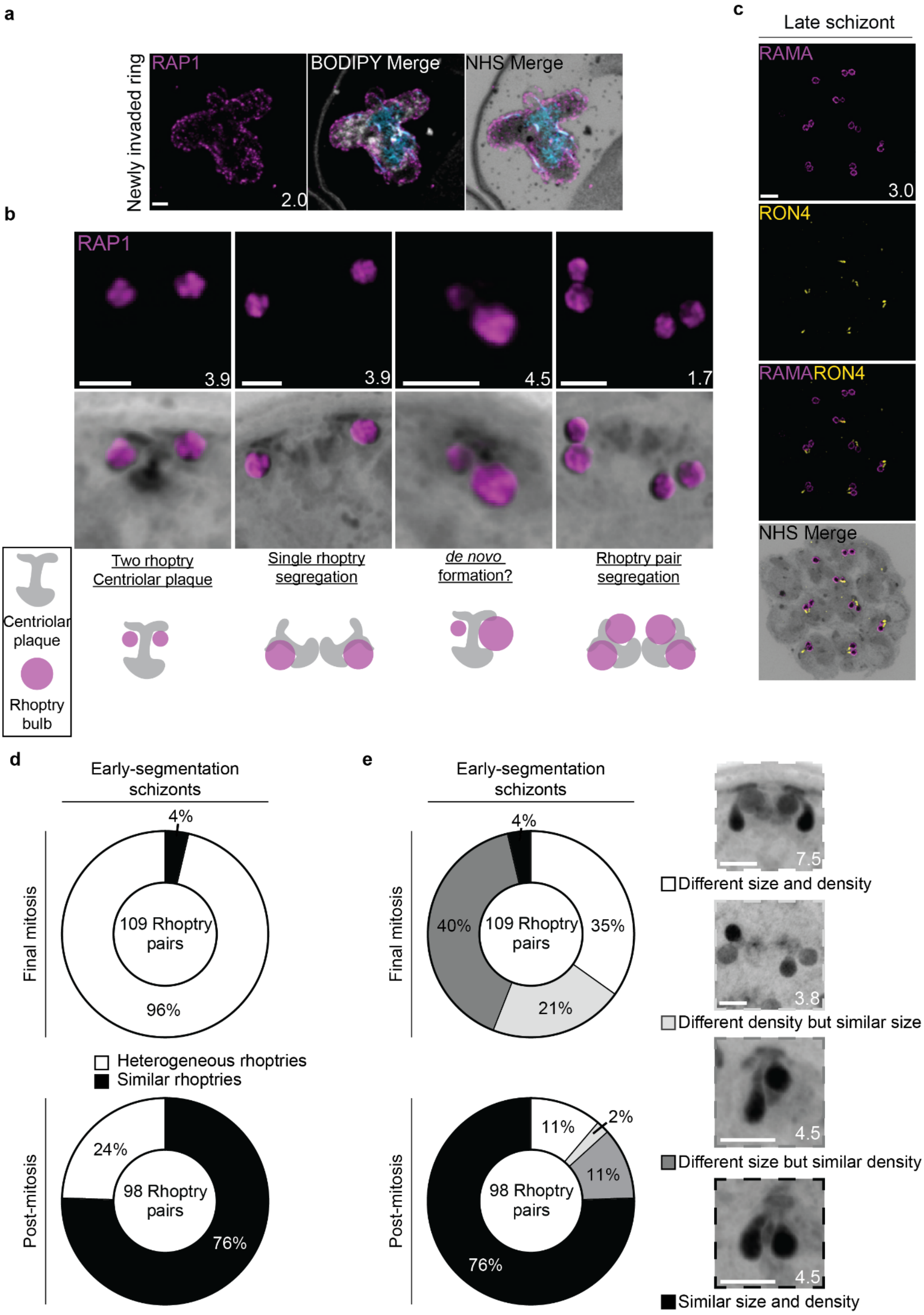

Figure 8 – Figure Supplement 1
