# Supplementary material for: Atlas of Plasmodium falciparum intraerythrocytic development using expansion microscopy
Source: bioRxiv. 2023 May 15:2023.03.22.533773. Originally published 2023 Mar 24. Preprint. [Version 2] doi: 10.1101/2023.03.22.533773 (PMC10055389; doi:10.1101/2023.03.22.533773)
Supplement: Supplementary file 1 [file media-1.pdf]

Atlas of *Plasmodium falciparum* intraerythrocytic development using expansion microscopy

SUPPLEMENTARY INFORMATION

Oligo/gBlock Name	Sequence (5’→3’)
oJDD44	TGGGGTGATGATAAAATGAAAG
oJDD56	ACACTTTATGCTTCCGGCTCGTATGTTGTG
oJDD4889	TATTGTCAAATCGTTACCTCTATG
oJDD4890	AAACCATAGAGGTAACGATTTGAC
oJDD4891	TAGgcggccgcGGTCCTACACCAATAAATATCA
oJDD4892	GTCTGATTCTTCCCATCaggccttccggaccgcggGGTCCCTTCATTGTAGACTTTTTATTATTGAAC
oJDD4893	GTCTACAATGAAGGGACCccgcggtccggaaggcctGATGGGAAGAATCAGACAAATGGT
oJDD4894	GATctcgagcAGcGGcAAtGAcTTcACgAATTCTCTTATTTCTTGTTTTTGCATTTCCT
oSAB257	CGGACCGAGAATTTATGTCCATTAACGTC
oSAB471	TGGTATTAATGGATGAAGACACACA
oSAB472	GTAATGGAATAGCTTTATATATGTACCTTCAT
oSAB473	TATGTGATCCATACATACCTGTTCAGAC

Supplementary Table 1: Oligonucleotides for cloning and integration PCR.

Primary Antibodies	Ab species	Antibody source (cat. Number)	Ab conc.	Reference
anti-alpha tubulin (Clone B-5-1-2)	Mouse (IgG1)	ThermoFisher (32-2500)	1:500	
anti-centrin (Clone 20H5)	Mouse (IgG2a)	Sigma-Aldrich (04-1624)	1:200	
anti-*Hs*centrin1	Rabbit	ThermoFisher (PA5-29986)	1:500	
anti-polyE (IN105)	Rabbit	Adipogen (AG-25B-0030-C050)	1:500	
anti-ERD2 (MRA-1)	Rabbit	BEI Resources MR4	1:2000	[1]
anti-HA (3F10)	Rat	Roche (12158167001)	1:50	
anti-GFP	Rabbit	OriGene (TP401)	1:2000	
anti-RAP1 (2.29)	Mouse	European Malaria Reagent Repository	1:500	[2]
anti-RON4	Mouse	Gift from Alan Cowman	1:100	
anti-RAMA	Rabbit	Gift from Ross Coppel	1:200	[3]
anti-AMA1	Rabbit	Gift from Carole Long	1:500	
anti-EBA175 (3D7)	Mouse	Gift from Alan Cowman	1:500	[4]
anti-Aldolase	rabbit	Abcam (ab207494)	1:2000	
anti-Histone H3	rabbit	Abcam (ab1791)	1:1000	
anti-BIP	rabbit	Generated by Dvorin Lab	1:2000	
anti-GAP45	Rabbit	Gift from Julian Rayner	1:2000	[5]
anti-IMC1g	Rabbit	Generated by Dvorin Lab	1:1000	
anti-MSP1 (1E1)	Rabbit	Gift from Anthony Holder	1:250	[6]
anti-Haemoglobin	Rabbit	ThermoFisher (PA5-102943)	1:1000	

Secondary antibodies	Antibody species	Antibody source	Antibody concentration
anti-mouse IgG Alexa Fluor 488	Goat	ThermoFisher (A28175)	1:500
anti-mouse IgG Alexa Fluor 555	Goat	ThermoFisher (A21428)	1:500
anti-rabbit IgG Alexa Fluor 488	Goat	ThermoFisher (A11034)	1:500
anti-rabbit IgG Alexa fluor 555	Goat	ThermoFisher (A21428)	1:500
anti-rat IgG Alexa fluor 488	Goat	ThermoFisher (A11006)	1:500
anti-mouse IgG2a Alexa fluor 488	Goat	ThermoFisher (A21131)	1:500
anti-mouse IgG1 Alexa fluor 594	Goat	ThermoFisher (A21125)	1:500

Stains	Stain source (Cat. No)	Stain concentration
NHS Ester Alexa fluor 405	ThermoFisher (A30000)	1:250 (8 μM in DMSO
BODIPY TR-Ceramide	ThermoFisher (D7549)	1:500 (2 μM)
SYTOX Deep Red	ThermoFisher (S11381)	1:1000 (1 μM) in DMSO

Supplementary Table 2: Summary of all antibodies and stains used in this study.

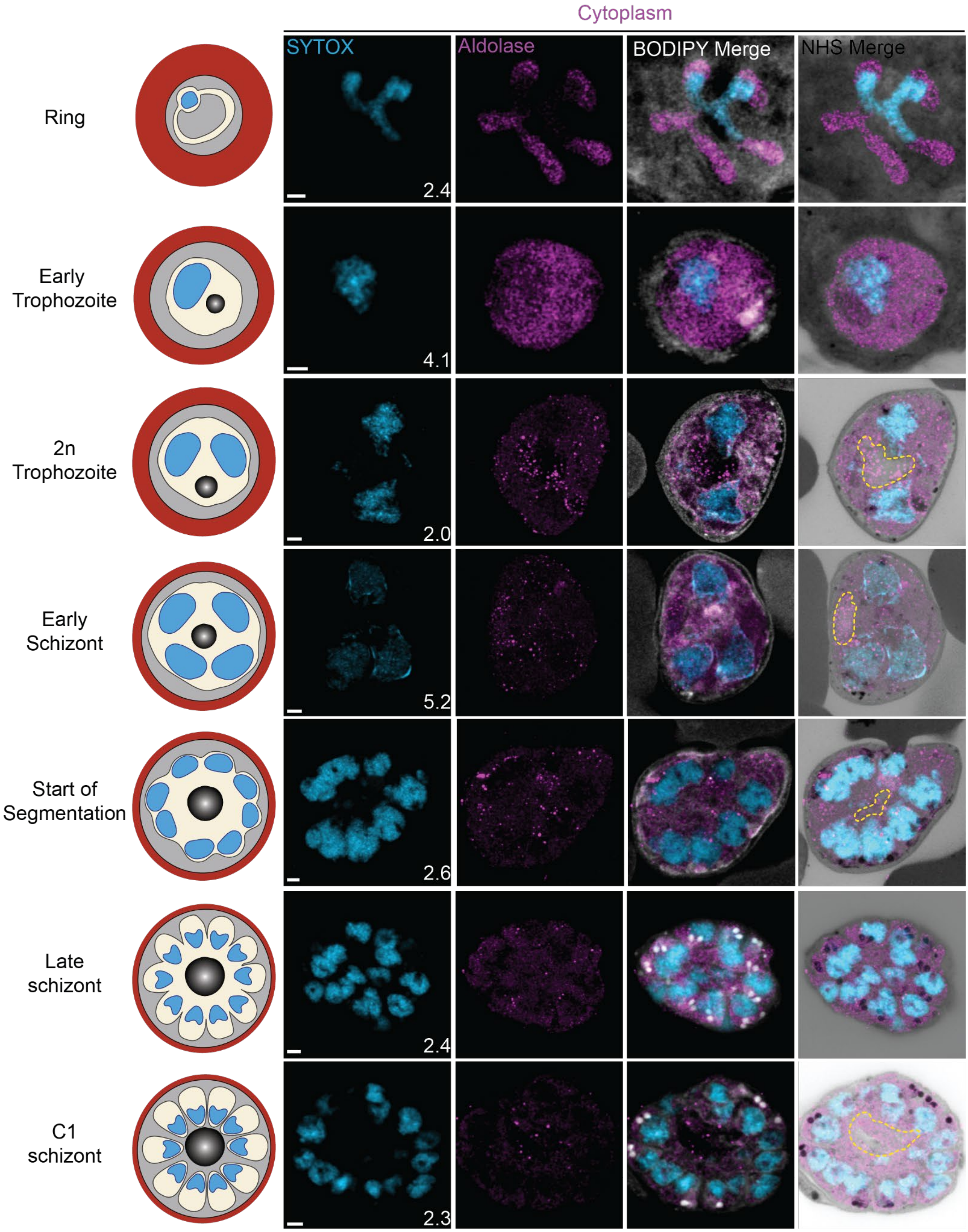

Supplementary Figure 1: Cytoplasm staining during intraerythrocytic development.
3D7 parasites were prepared by U-ExM, stained with NHS Ester (greyscale), BODIPY TRc (white), SYTOX (cyan) and anti-aldolase (cytoplasm; magenta) antibodies and imaged by Airyscan microscopy across the asexual blood-stage. Yellow line indicates likely position of food vacuole lacking hemozoin crystal. Images are maximum-intensity projections, number on image = Z-depth in µm of projection. Scale bars = 2 µm.

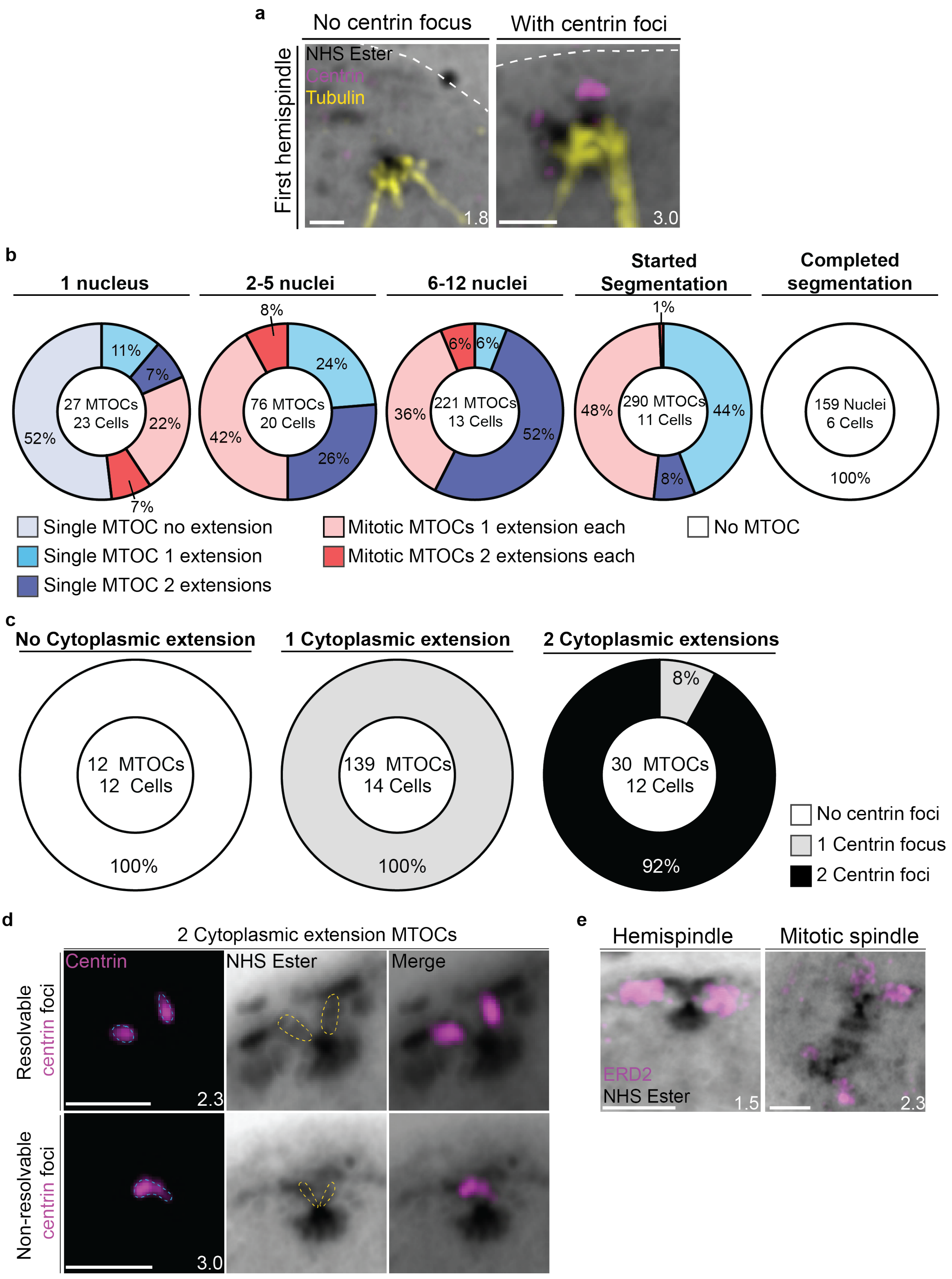

Supplementary Figure 2: Characterisation of MTOC cytoplasmic extensions.

(a) *Mononucleated 3D7 parasites were prepared by U-ExM, stained with NHS Ester (greyscale), anti-centrin (MTOC, magenta) and anti-tubulin (microtubules, yellow) antibodies and imaged by Airyscan microscopy. Nuclei form the first hemispindle before centrin is observed, and associate with the parasite plasma membrane (PPM) following visualisation of centrin.* ***(b)*** *Quantification of the number of cytoplasmic extensions per MTOC in parasites of varying age. Indvidual MTOCs from mitotic spindles were assessed separately.* ***(c)*** *Quantification of the co-occurrence of cytoplasmic extensions with centrin foci.* ***(d)*** *In the 8% of 2 cytoplasmic extension MTOCs that had only a single centrin focus, these were likely non-resolvable at the image resolution.* ***(e)*** *Parasites that had started mitosis were stained with NHS Ester and Golgi marker ERD2 (magenta). Golgi was seen associating with MTOCs and appeared to duplicate with the formation of the mitotic spindle. Images are maximum-intensity projections, number on image = Z-depth in µm of projection. Scale bars = 2 µm.*

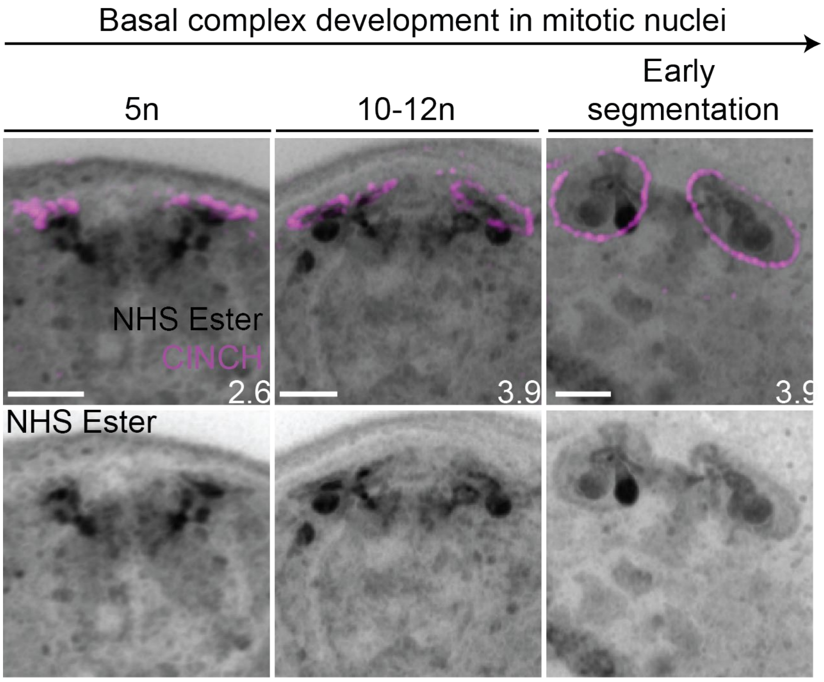

Supplementary Figure 3: Basal complex biogenesis in mitotic nuclei.

Parasites expressing an smV5-tagged copy of the basal complex marker CINCH were prepared by U-ExM, stained with NHS Ester (greyscale), and anti-V5 (basal complex; magenta) antibodies and imaged by Airyscan microscopy across segmentation. All nuclei shown in Figure 4b, which describes basal complex biogenesis, had a single MTOC. This figure shows comparable images of nuclei that have duplicated their MTOC and have a visible mitotic spindle. Images are maximum-intensity projections, number on image = Z-depth in µm of projection. Scale bars = 2 µm.

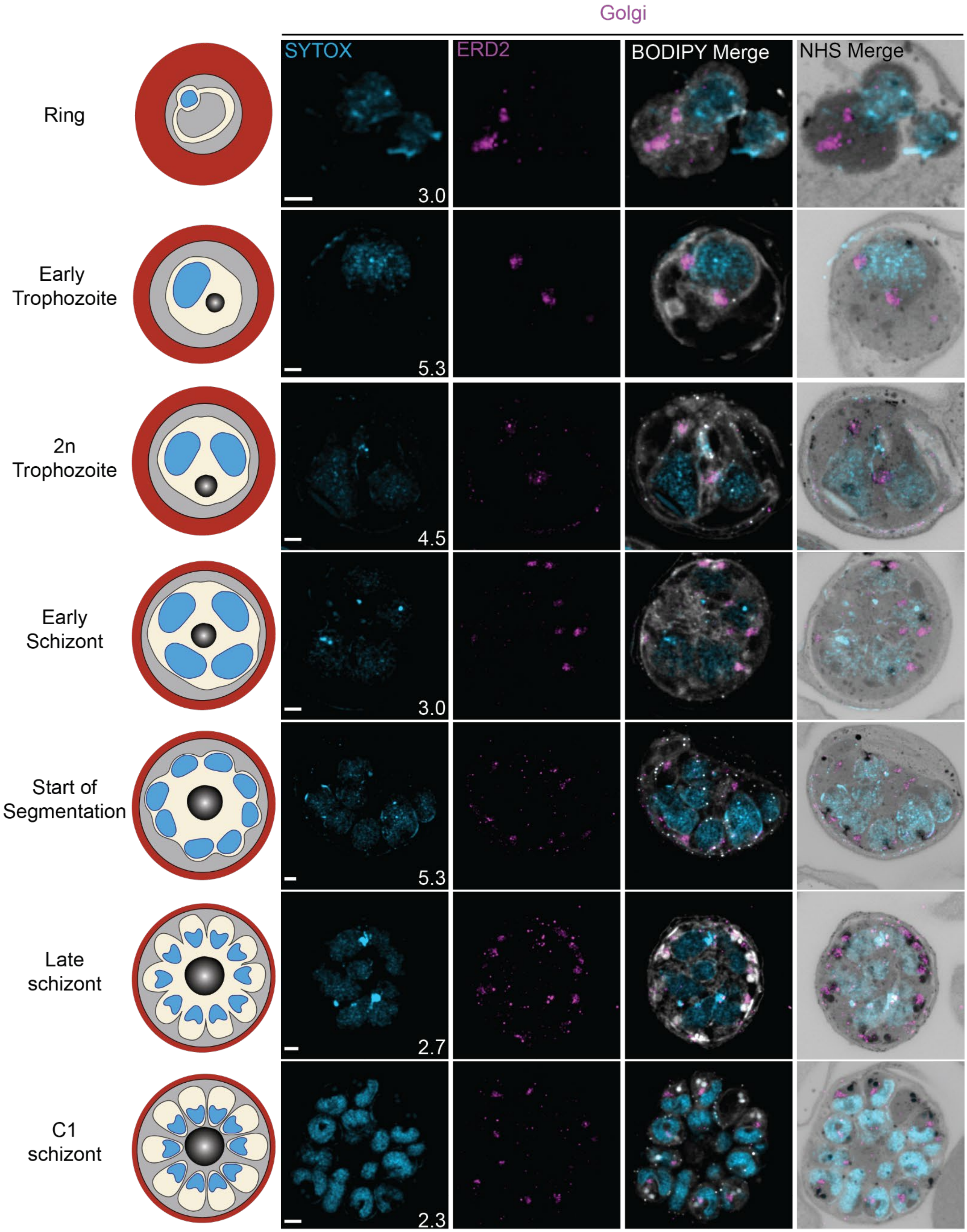

Supplementary Figure 4: Golgi staining during intraerythrocytic development.

3D7 parasites were prepared by U-ExM, stained with NHS Ester (greyscale), BODIPY TRc (white), SYTOX (cyan) and anti-ERD2 (Golgi; magenta) antibodies and imaged by Airyscan microscopy across the asexual blood-stage. Images are maximum-intensity projections, number on image = Z-depth in µm of projection. Scale bars = 2 µm.

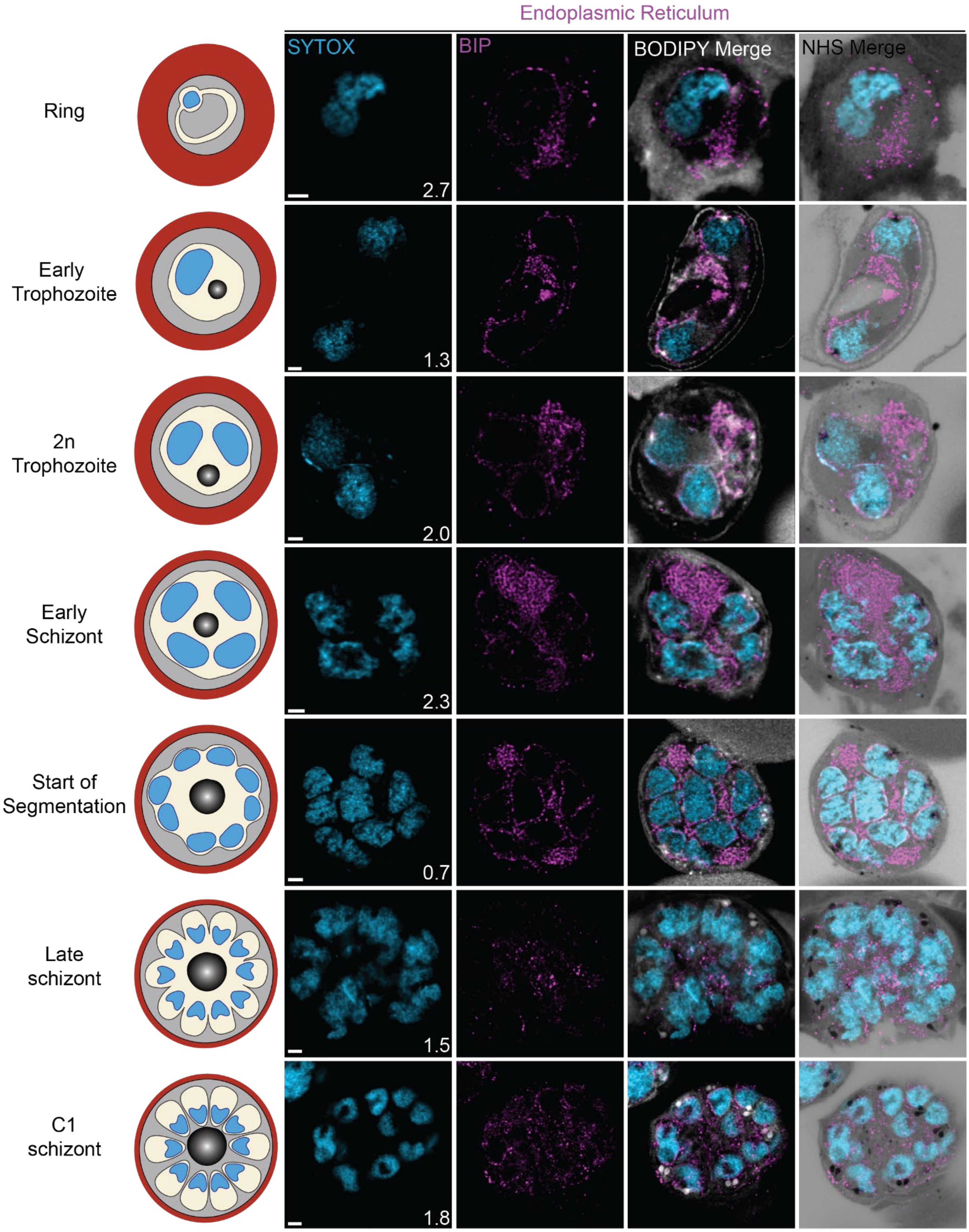

Supplementary Figure 5: Endoplasmic reticulum staining during intraerythrocytic development.

3D7 parasites were prepared by U-ExM, stained with NHS Ester (greyscale), BODIPY TRc (white), SYTOX (cyan) and anti-BIP (Endoplasmic reticulum; magenta) antibodies and imaged by Airyscan microscopy across the asexual blood-stage. Images are maximum-intensity projections, number on image = Z-depth in µm of projection. Scale bars = 2 µm.

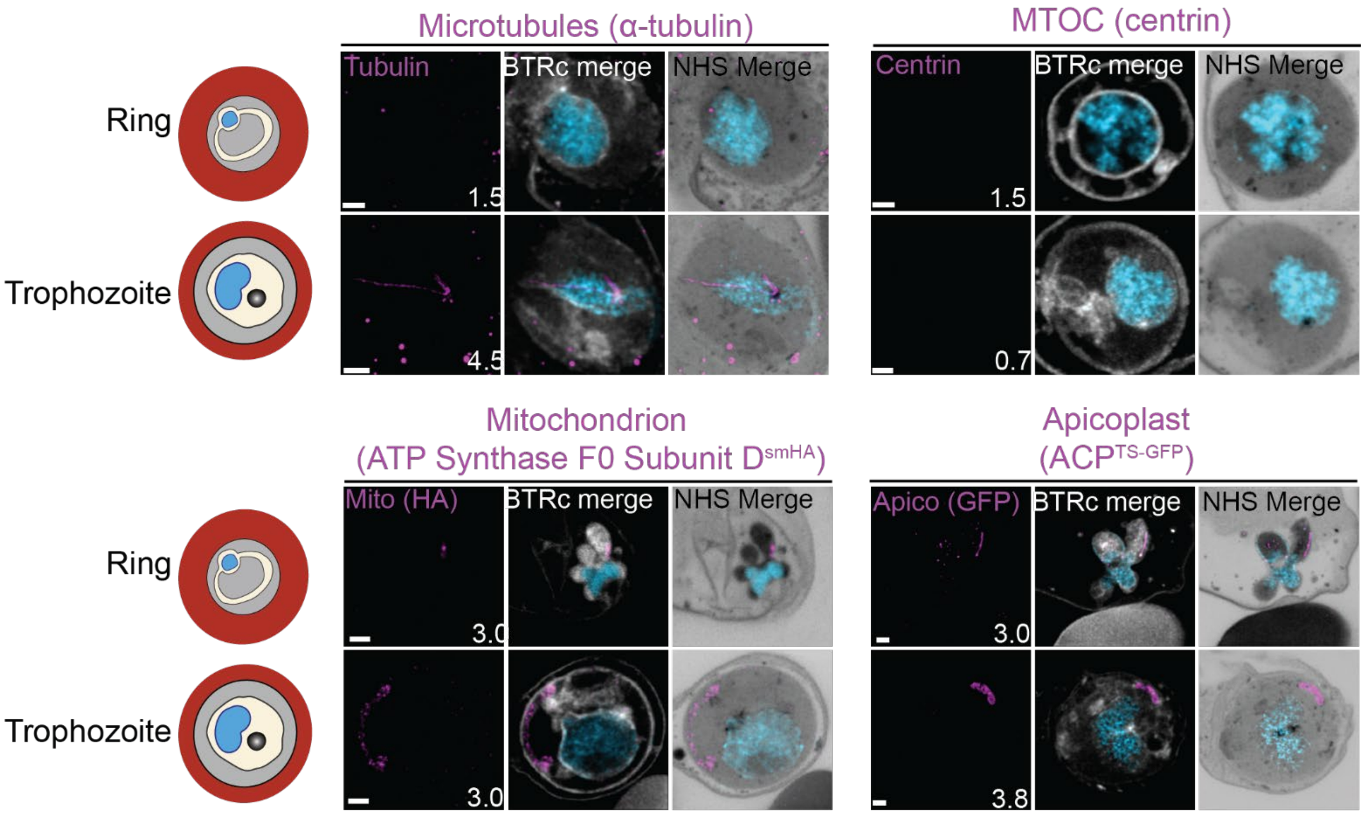

Supplementary Figure 6: Staining of microtubules, MTOC, mitochondrion and apicoplast in rings or early trophozoite stages.

The focus of this study was parasites undergoing schizogony, but multiple ring-stage parasites and mononucleated trophozoites were also imaged. For microtubules and centrin, 3D7 parasites were prepared by U-ExM, stained with NHS Ester (greyscale), BODIPY TRc (white), SYTOX (cyan) and either anti-tubulin (microtubules) or anti-centrin (MTOC) (magenta) antibodies and imaged using Airyscan microscopy. For the mitochondrion, ATP synthase F0 subunit D^{smHA} parasites were stained as described for microtubules and MTOC except with anti-HA (mitochondrion) antibodies. For the apicoplast, parasites expressing a GFP-tagged copy of the apicoplast targeting signal of acyl carrier protein were stained as described previously except with anti-GFP (apicoplast) antibodies. Images are maximum-intensity projections, number on image = Z-depth in µm of projection. Scale bars = 2 µm.

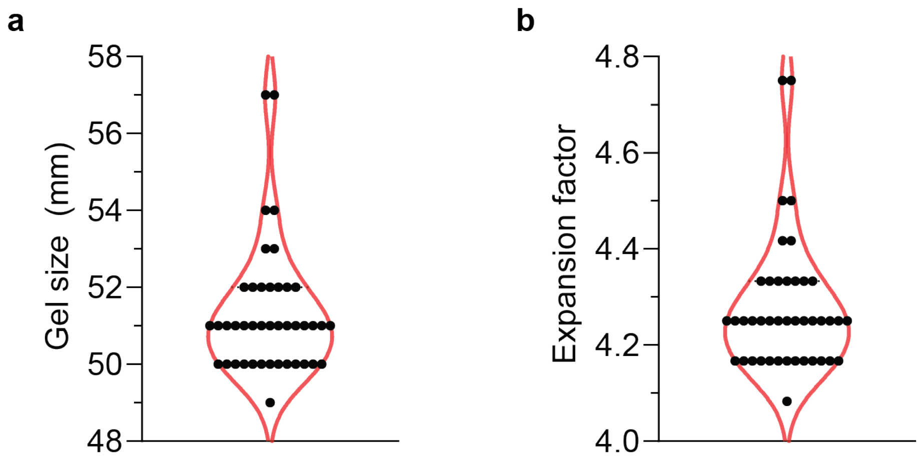

Supplementary Figure 7: Size of gels imaged in this study.

(a) *42 of the expanded gels in this study were measured post-expansion to* ***(b)*** *calculate their expansion factor****.*** *The median gel length was 51 mm, which corresponds to a 4.25x expansion factor, and so for 'actual' measurements, values were divded by 4.25.*

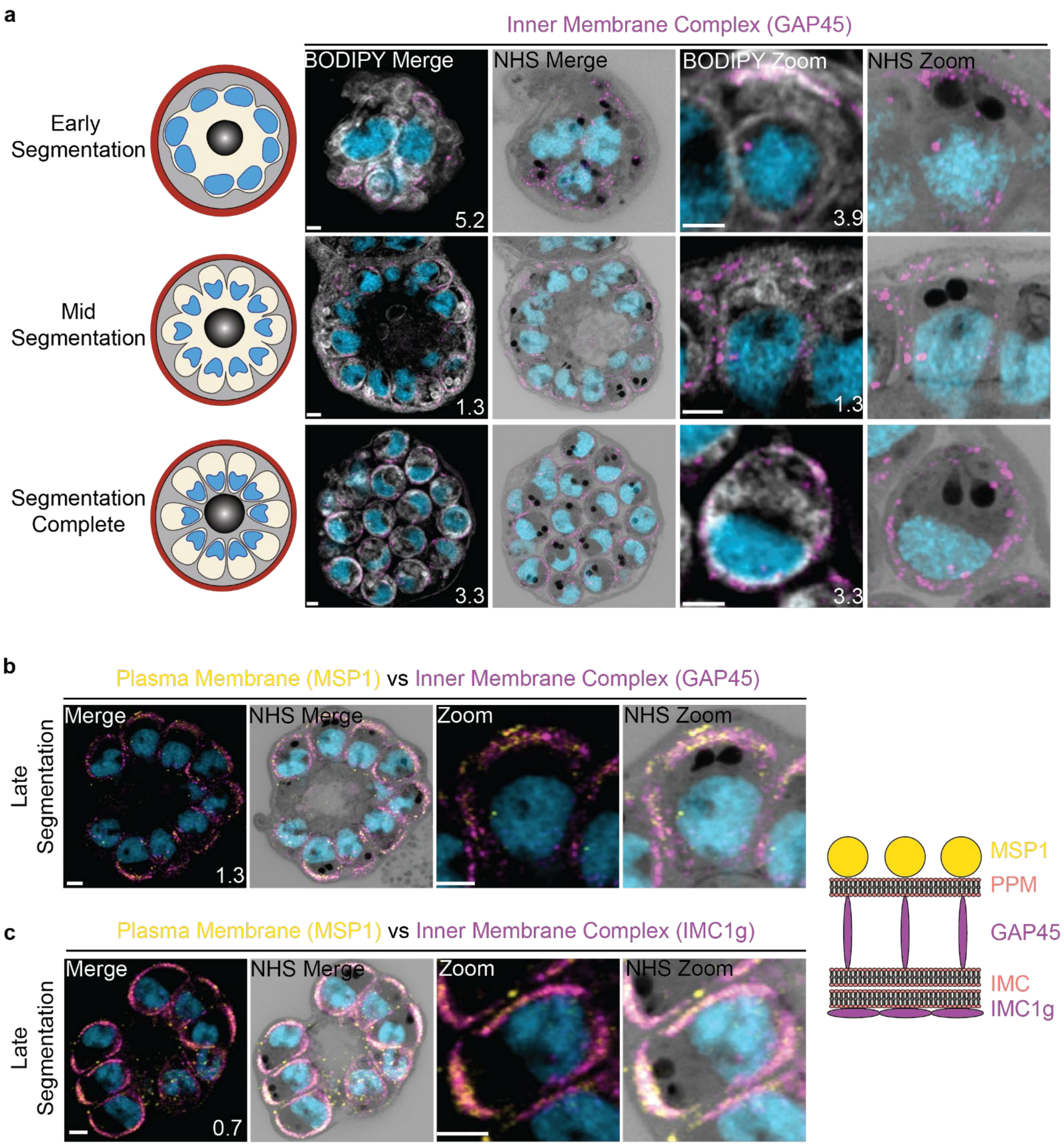

Supplementary Figure 8: Inner membrane complex progression through segmentation.

(a) *3D7 parasites were prepared by U-ExM, stained with NHS Ester (greyscale), BODIPY TRc (white), SYTOX (cyan) and anti-GAP45 (IMC) (magenta) antibodies and imaged using Airyscan microscopy across segmentation. 3D7 parasites were stained with NHS ester, SYTOX, anti-MSP1 (parasite plasma membrane) and either anti-GAP45* ***(b)*** *or anti-IMC1g* ***(c)*** *antibodies. GAP45 resides in the IMC luminal space, while IMC1g resides on the cytosolic side of the IMC. Neither GAP45 or IMC1g could be reliably distinguished from MSP1. Images are maximum-intensity projections, number on image = Z-depth in µm of projection. Scale bars = 2 µm.*

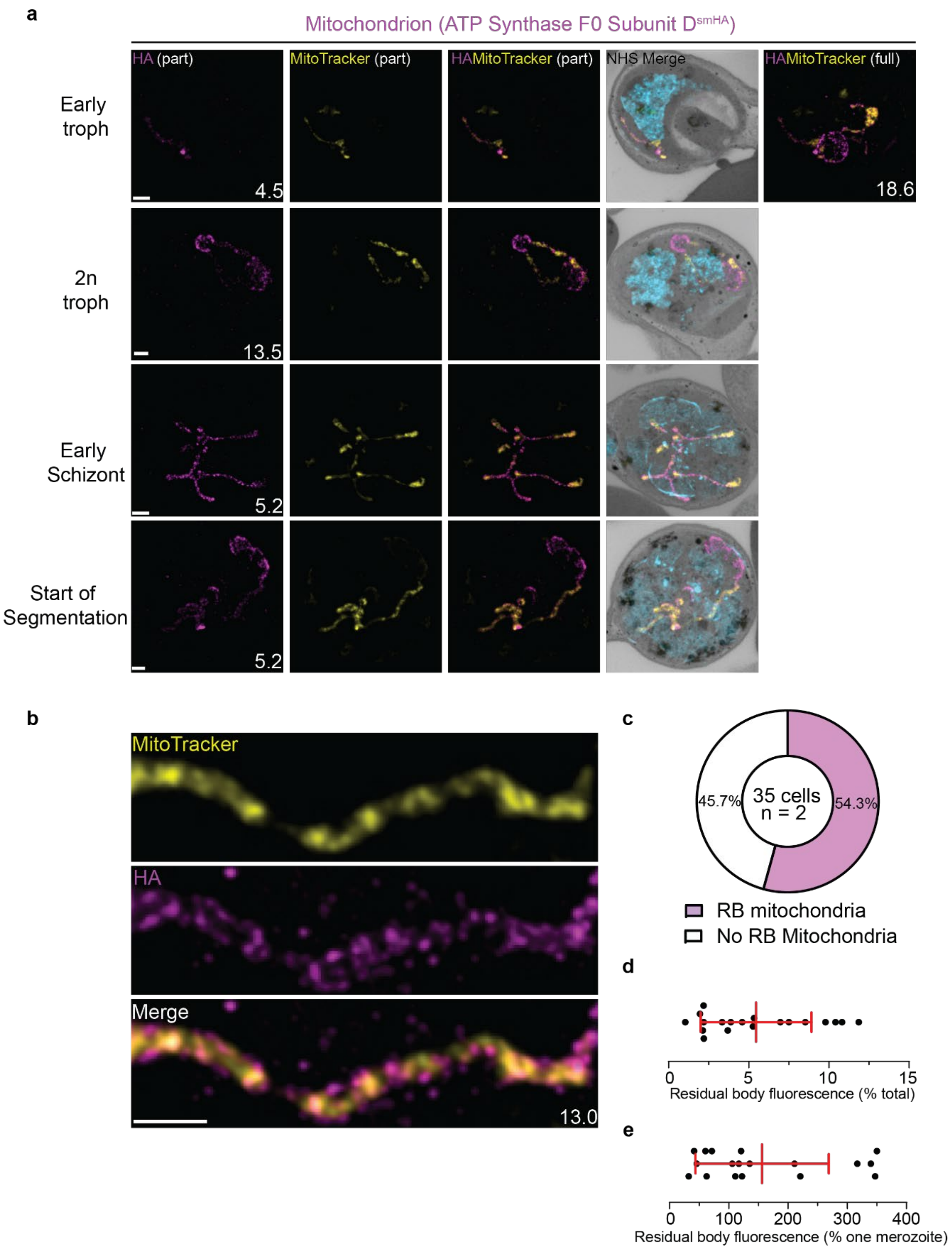

Supplementary Figure 9: Comparison of MitoTracker with ATPd staining, and residual body mitochondria quantification.

(a) *Parasites with an smHA-tagged copy of the ATP Synthase F0 Subunit D (Pf3D7_0311800), a mitochondrial marker, were prepared by U-ExM, stained with NHS Ester (greyscale), BODIPY TRc (white), SYTOX (cyan), Mitotracker (mitochondrion; yellow) and anti-HA (mitochondrion; magenta) antibodies and imaged Airyscan microscopy across the asexual blood-stage. Maximum intensity projections that showed NHS-Ester, BODIPY and SYTOX of discernible structures often did not give a good indication of mitochondria shape. To address this, projections of both a subsection of the cell (part) and the full cell (full) are shown.* ***(b)*** *MitoTracker staining showed numerous punctae along the mitochondrion.* ***(c)*** *The percentage of C1-arrested schizonts where mitochondria staining was observed in the residual body (RB) was quantified. This fluorescence was then compared against the total mitochondria fluoresence* ***(d)*** *and the mean mitochondria fluorescence of one merozoite within that schizont* ***(e).*** *Images are maximum-intensity projections, number on image = Z-depth in µm of projection. Scale bars = 2 µm.*

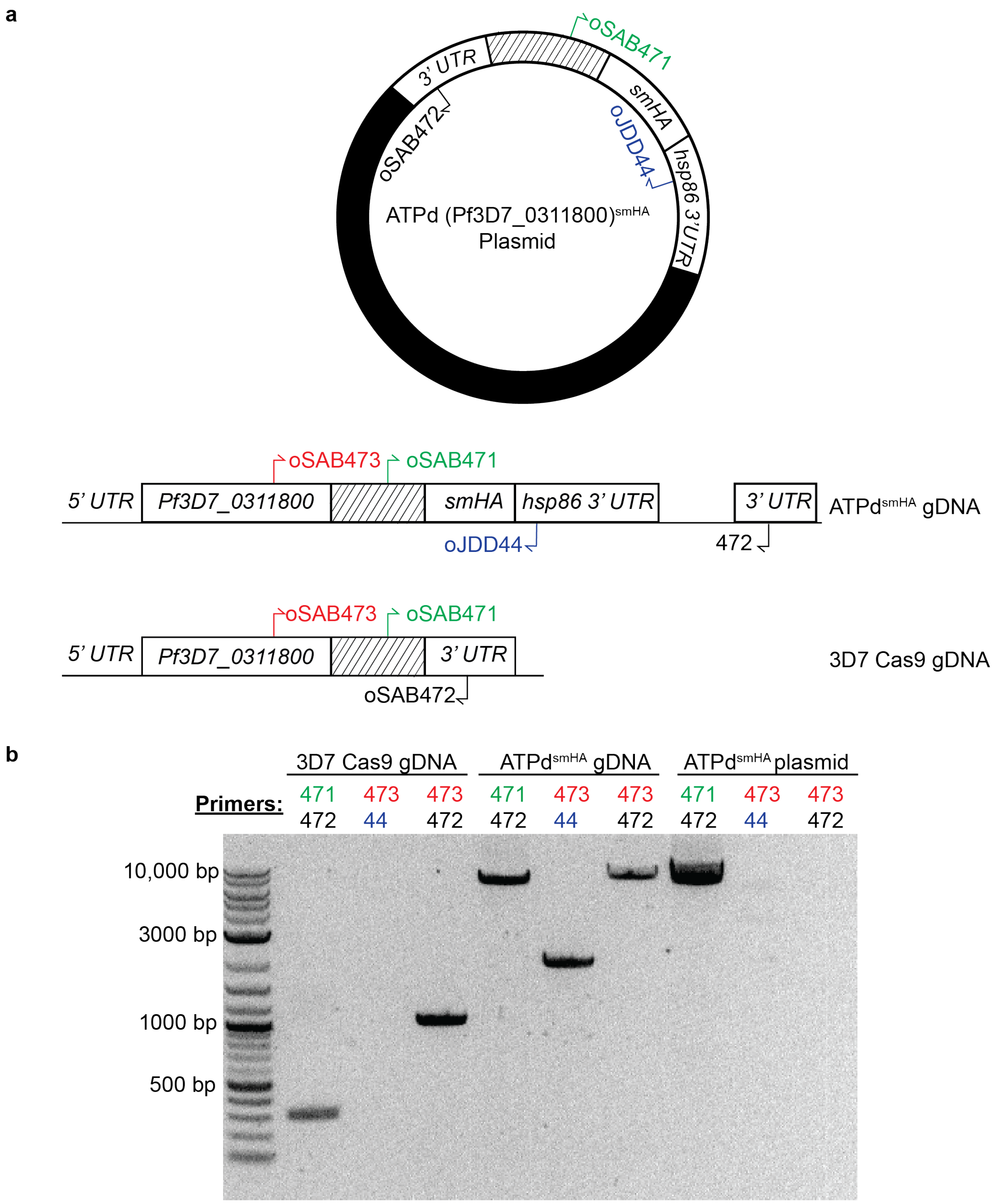

	471/472	473/44	473/472
3D7 Cas9 gDNA	321 bp	--------	1038 bp
ATPd^{smHA} gDNA	6890 bp	2017 bp	8008 bp
ATPd^{smHA} plasmid	6890 bp	--------	--------

Supplementary Figure 10: Generation of ATPd (Pf3D7_0311800) smHA parasites.

(a) *To generate an smHA-tagged copy of ATPd (Pf3D7_0311800), 3D7 Cas9 parasites were transfected with a plasmid that contained a 3' homology region to ATPd, followed by a C-terminal smHA-tag.* ***(b)*** *Integration of smHA tag into the ATPd locus was confirmed by PCR.*

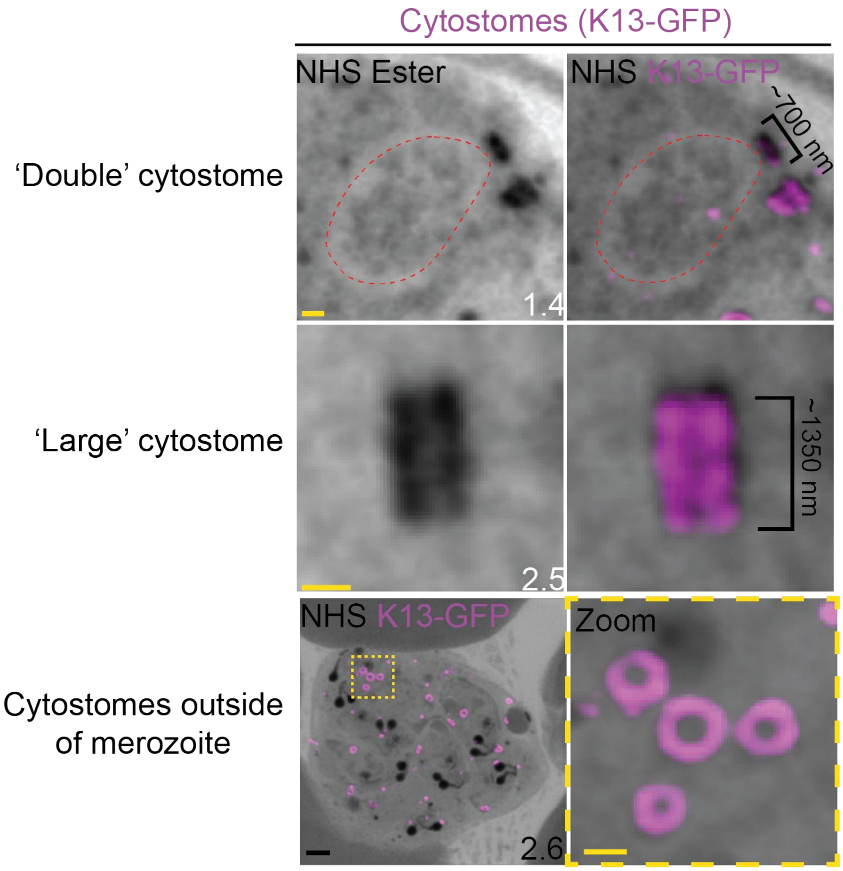

Supplementary Figure 11: Observed cytostome morphologies.

Parasites expressing a GFP-tagged copy of the cytostome marker Kelch13 (K13-GFP) were prepared for U-ExM, stained with NHS Ester (greyscale) and anti-GFP (cytostome; magenta) antibodies and imaged using Airyscan microscopy. 'Double' cytostomes where two collars appeared to be stacked on top of each other, and cytostomes approximately twice the diameter of other cytostomes were occasionally observed. Additionally, in almost all segmenting schizonts, multiple cytostomes were observed that were not incorporated into the forming merozoites. Images are maximum-intensity projections, number on image = Z-depth in µm of projection. Black scale bar = 2 µm, yellow scale bars = 500 nm.

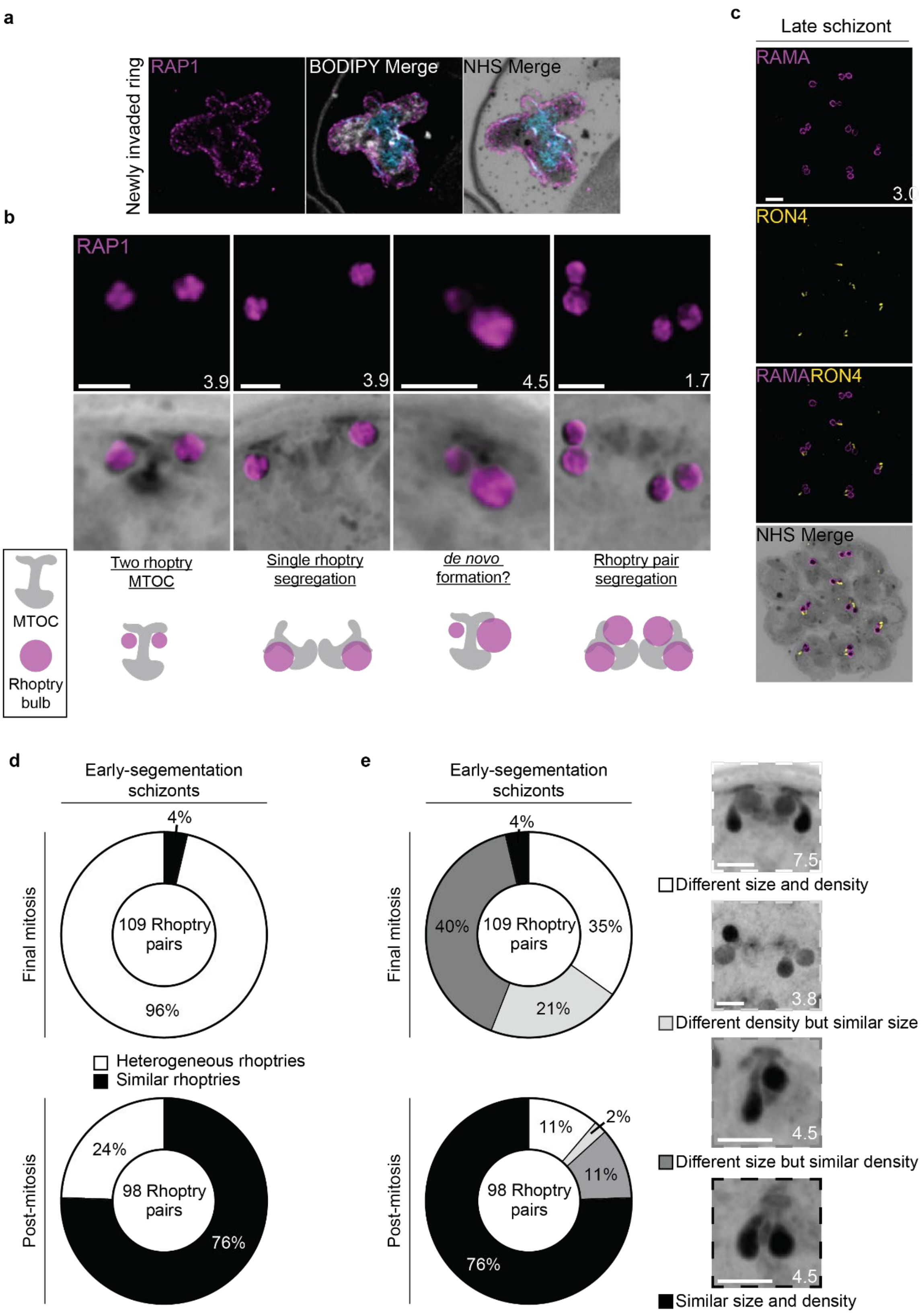

Supplementary Figure 12: Rhoptry biogenesis during schizogony.

(a) *The rhoptry marker RAP1 was observed coating the outside of a recently invaded ring-stage parasite.* ***(b)*** *Different combinations of rhoptries and MTOCs observed while imaging parasites undergoing schizogony.* ***(c)*** *Representative image of E64-arrested schizont stained with NHS Ester (greyscale), the rhoptry bulb marker RAMA (magenta) and the rhoptry neck marker RON4 (yellow).* ***(d)*** *In early-segmentation schizonts that were either undergoing their final mitosis, or had completed their final mitosis (post-mitosis), the degree of hetereogeneity between rhoptry pairs was quantified. Rhoptry pairs that showed visible differences in either size or NHS ester staining density were considered heterogeneous.* ***(e)*** *Breakdown of rhoptries using different classifications of hereogeneity. Images are maximum-intensity projections, number on image = Z-depth in µm of projection. Scale bars = 2 µm.*

SUPPLEMENTARY REFERENCES

1. Elmendorf, H.G. and K. Haldar, *Identification and localization of ERD2 in the malaria parasite Plasmodium falciparum: separation from sites of sphingomyelin synthesis and implications for organization of the Golgi.* The EMBO Journal, 1993. **12**(12): p. 4763-4773.
2. Hall, R., et al., *Antigens of the erythrocytes stages of the human malaria parasite Plasmodium falciparum detected by monoclonal antibodies.* Mol Biochem Parasitol, 1983. **7**(3): p. 247-65.
3. Topolska, A.E., et al., *Characterization of a membrane-associated rhoptry protein of Plasmodium falciparum.* J Biol Chem, 2004. **279**(6): p. 4648-56.
4. Sim, B.K.L., et al., *Delineation of Stage Specific Expression of Plasmodium falciparum EBA-175 by Biologically Functional Region II Monoclonal Antibodies.* PLOS ONE, 2011. **6**(4): p. e18393.
5. Jones, M.L., C. Cottingham, and J.C. Rayner, *Effects of calcium signaling on Plasmodium falciparum erythrocyte invasion and post-translational modification of gliding-associated protein 45 (PfGAP45).* Molecular and Biochemical Parasitology, 2009. **168**(1): p. 55-62.
6. Blackman, M.J., et al., *Antibodies inhibit the protease-mediated processing of a malaria merozoite surface protein.* Journal of Experimental Medicine, 1994. **180**(1): p. 389-393.
